# Variation in microbial and plant associations across wild bee families, functional groups, and urban environments

**DOI:** 10.1093/femsec/fiag109

**Published:** 2026-09-30

**Authors:** Katherine D Chau, Trevor J L Sless, Phuong N Nguyen, Sandra M Rehan

**Affiliations:** Department of Biology, York University, Toronto, ON M3J 1P3, Canada; Department of Biology, York University, Toronto, ON M3J 1P3, Canada; Department of Biology, York University, Toronto, ON M3J 1P3, Canada; Department of Biology, York University, Toronto, ON M3J 1P3, Canada

**Keywords:** microbiome, plant associations, bacteria, fungi, wild bees, urbanization

## Abstract

Bees harbour diverse microbiomes and floral associations, which may shift in response to several biological and environmental factors. We examined the diversity of bacterial, fungal, and plant associates (i.e. from foraging or nesting material) across 32 bee species from five families (Apidae, Megachilidae, Halictidae, Colletidae, and Andrenidae) using 16S rRNA, ITS1, and rbcL amplicon sequencing. The included bee species represent multiple axes of variation across functional traits including body size, nesting behaviour, sociality, diet, and native status, and response to urbanization. Apidae, Colletidae, and Andrenidae bee families had richer communities of bacterial and plant associates than Halictidae and Megachilidae, while fungal diversity and composition were similar across families. Common microbes and plant associations were detected across the bee community, but certain taxa were common only to specific bee species. Plant associations more often shifted significantly in diversity depending on functional trait and urban intensity, whereas bacterial and fungal diversity were largely consistent across bee families, functional traits, and urban intensities. Our findings show that while microbial communities are fairly stable across bee lineages and functional groups, bee-associated plant communities vary with urbanization and are the primary factor differentiating bee species in urban landscapes.

## Teaser

Microbial communities are stable across bee lineages and functional groups, bee-associated plants are sensitive to environmental variation and serve as a primary axis that differentiates bee species in urban landscapes.

## Background

With over 20 000 described species spanning seven families, bees (Hymenoptera: Anthophila) are an incredibly diverse group of insects that provide essential pollination services which contribute to global food security (Khalifa et al. 2021). Bees exhibit remarkable ecological and evolutionary diversity, encompassing multiple distinct functional guilds that reflect the unique roles bees play in many ecosystems. Correspondingly, bees possess diverse microbiomes (e.g. bacteria and fungi that reside within or on the host) which can vary according to the host’s taxonomic affinities, their social behaviour, or even their ecological niche (Kwong et al. 2017, Hammer et al. 2021). Increasing evidence show that bee-associated microbial communities influence host health and fitness, thus, understanding the factors that structure these microbiomes has become an important area of research. Acquisition of microbes differs greatly depending on various characteristics of bees. For example, the social behaviour of bees has been shown to impact acquisition of microbes, such that eusocial bee microbiomes are influenced primarily by social interactions with colony members (Kwong and Moran 2016), whereas solitary and subsocial bee microbiomes are shaped by interactions with the environment (McFrederick and Rehan 2016, Dew et al. 2020). Owing to their reduced exposure with conspecifics, solitary bees host more diverse bacteria compared to their eusocial counterparts and their microbiomes are typically dominated by environmental symbionts found in soil or flowers (Thamm et al. 2023, Hettiarachchi et al. 2023).

Convergently evolved characteristics allow certain bee species to exploit niche plant resources and maximize their nutritional gain. For example, buzz pollination helps extract inaccessible pollen from flowers with poricidal anthers and evolved almost 45 times independently in bee genera in almost all bee families, thus leading to plant associations due to foraging on plant families with poricidal anthers such as Solanaceae, Melastomataceae, and Fabaceae (Cardinal et al. 2018, Mesquita-Neto et al. 2018). Some bee species are more specialized (i.e. pollen specialist) and have reduced plant associations as a result of exclusively feeding on specific flower families or genera. These relationships have critical implications for maintaining healthy bee populations, especially for specialists when their limited nutritional resources are more easily altered by habitat changes (Anderson et al. 2024).

In addition to sociality and diet, other bee traits such as body size, nesting behaviour, and whether the bee is native or introduced also contribute to variation of microbiota and floral associations. The influence of body size of the bee species on microbial communities remains understudied, but some studied suggest that body size could play a role in shaping microbial communities. For instance, in some regions larger bee species were found to have a more specialized diet (Raiol et al. 2021), and would therefore have reduced floral associations than the smaller, generalist bee species. In contrast, larger bees are capable of foraging longer distances and, in some cases, will forage farther for better floral rewards (Greenleaf et al. 2007), thus increasing their contact with diverse floral resources and environmental microbes. Differences in nesting substrate may also shape microbial acquisition, as ground-nesting bees are exposed to soil-associated microbes, while cavity nesting bees are exposed to plant-associated microbes either through the phyllosphere (i.e. exposed plant tissue) or endosphere (i.e. internal plant tissue) (Turner et al. 2013). Variation within the nesting substrate, such as differences in soil physicochemical properties or type of plant nesting material, also impact the microbial community composition and abundance (de Sousa 2021, Bogati et al. 2025). Native status of bees also influences the susceptibility and tolerance of microbial exposure as well as the preference of foraging patterns. Introduced bees have not coevolved with the native flora nor native environmental symbionts, and would thus harbor a different microbiome composition and plant-association compared to native species in the same area (Pei et al. 2023). Native status was also found to impact the prevalence of parasitic microbes in bees in which introduced bee species (often managed species) act as parasite reservoirs, transmitting parasitic microbes onto plants that spillover onto native bee populations, often as a detriment to the native species (Melanson et al. 2026, Motta et al. 2026).

Bee functional traits further interact with varying degrees of urbanization to alter host–taxon relationships. Major land use shifts caused by human activity disrupt natural bee habitats and native floral resources and show no signs of slowing down (Matteson et al. 2012, Ayers and Rehan 2021). Nonetheless, certain traits can increase bee species’ robustness against the negative effects of urbanization. Some studies have generalized that small-bodied bees, cavity-nesters, social bees, generalists, and introduced bee species thrive better in cities due to their greater tolerance to urban phenomena, such as elevated temperatures, phenological mismatch, and reduced or altered plant diversity (Fitch et al. 2019, Wilson and Jamieson 2019, Stemkovski et al. 2020, Ferrari et al. 2024). Thus, it is no surprise that urbanization impacts bee microbiomes and bee foraging preferences, since urbanization itself imposes stringent environmental filtering for bees with disadvantaged traits. In some instances, urbanization increased microbial richness in urban bees but with an increased risk of exposure to potential pathogenic microbes (Chau et al. 2023, Nguyen and Rehan 2022). In other instances, increasing urbanization decreases bacterial diversity in bee larvae (Peters et al. 2025).

Change in urban floral diversity alters bee–plant networks, which may lead to reduced bee abundance in cities for certain species whereas other species may exploit novel resources and better tolerate urbanization (Ayers and Rehan 2023, Brasil et al. 2024). Host–microbiota and host–plant interactions across bee families and bee traits serve as an important framework for highlighting ecological relationships that shape bee health and their resilience to environmental change.

Utilizing bee functional traits to understand how bee microbiomes and their preferred plant resources vary has been useful to discern trait-based patterns observed in an urban context (Wilson and Jamieson 2019, Ostwald et al. 2024). Here, we characterize the bacterial and fungal microbiome, and plant associations for bee species from five different bee families (Apidae, Megachilidae, Andrenidae, Colletidae, and Halictidae) sampled across an urban gradient and collected bees varying in functional traits. This study has two aims, with our first to assess the diversity of bacteria, fungi, and plant associates across bee species and bee functional traits, with the goal of determining both shared taxa among bees and unique taxa associated with specific bee species or functional traits. We predicted that taxa previously identified as common in bees such as the bacteria *Apilactobacillus* (Todorov et al. 2024), yeasts such as *Starmerella* and *Metschnikowia* (Rutkowski et al. 2023), or highly abundant plant genera in our sample region such as *Trifolium* will be shared across all our sampled bee species and traits. We also predicted that there will be host-specific and trait-specific taxa—taxa that are not as prevalent in the study but would be abundant in certain bee species or distinct functional traits—that contribute to variation in bee microbiome communities and plant-associate compositions. Second, we sought to determine how urbanization impacts the microbiome and plant associations across bee species and functional traits. Based on previous research, we expect increasing urbanization to decrease plant associations across bee species due to decreased floral resources, with a greater impact on specialists due to their restricted foraging patterns in urban environments.

## Methods

### Sample sites and bee sampling

We collected bees from 2022 to 2023 during late spring to late summer (May–September), sampling between 10:00 and 16:00. We established sampling across a gradient of urbanization levels within the Greater Toronto Area, Canada by selecting sites that covered a broad range of impervious surfaces within a 500 m radius buffer zone, which included ‘high’ urban sites (>80% impervious surface coverage), ‘medium’ urban sites (40%–80% impervious surface coverage), and ‘low’ urban sites (<40% impervious surface coverage). We calculated the impervious surface coverage using the Southern Ontario Land Resource Information System (SOLRIS) version 3.0 dataset (https://www.arcgis.com/home/item.html?id=0279f65b82314121b5b5ec93d76bc6ba) within QGIS version 3.36–2-Maidenhead (QGIS.org 2024). Impervious surfaces included *transportation* and *built-up area-impervious*. Other land type features included open water (*open water*), pervious surfaces (*pervious built-up, tallgrass woodland, forest, coniferous forest, mixed forest, deciduous forest, treed swamp, thicket swamp*, and *hedge rows*), and unclassified (*undifferentiated*). In total, 17 sites were chosen (6 high, 6 medium, and 5 low) (Fig. 1, Table S1). We collected bees from flowers via hand-netting and stored them in cryotubes in a cooler while in the field. We identified bees with keys (Mitchell 1960, 1962, Ascher and Pickering 2024) prior to flash-freezing them in liquid nitrogen for long-term storage at −80°C. Bee specimen species as well as their functional traits were recorded (see Table S1). We used the mean of the lower and upper female body size ranges obtained from literature to determine bee species’ body sizes; species are classified as ‘small’ if the mean length was 2–6 mm, ‘medium’ if the mean length was >6–10 mm, or ‘large’ if the mean length was >10 mm. Additional classifications included nesting behaviour (ground-nesters or cavity-nesters, which include stem nesting and snail shell nesting bee species), diet (generalists or specialists), sociality [solitary, subsocial (the mother remains at the nest and exhibit extended parental care), or eusocial], and native status (native or introduced).

**Figure 1:**
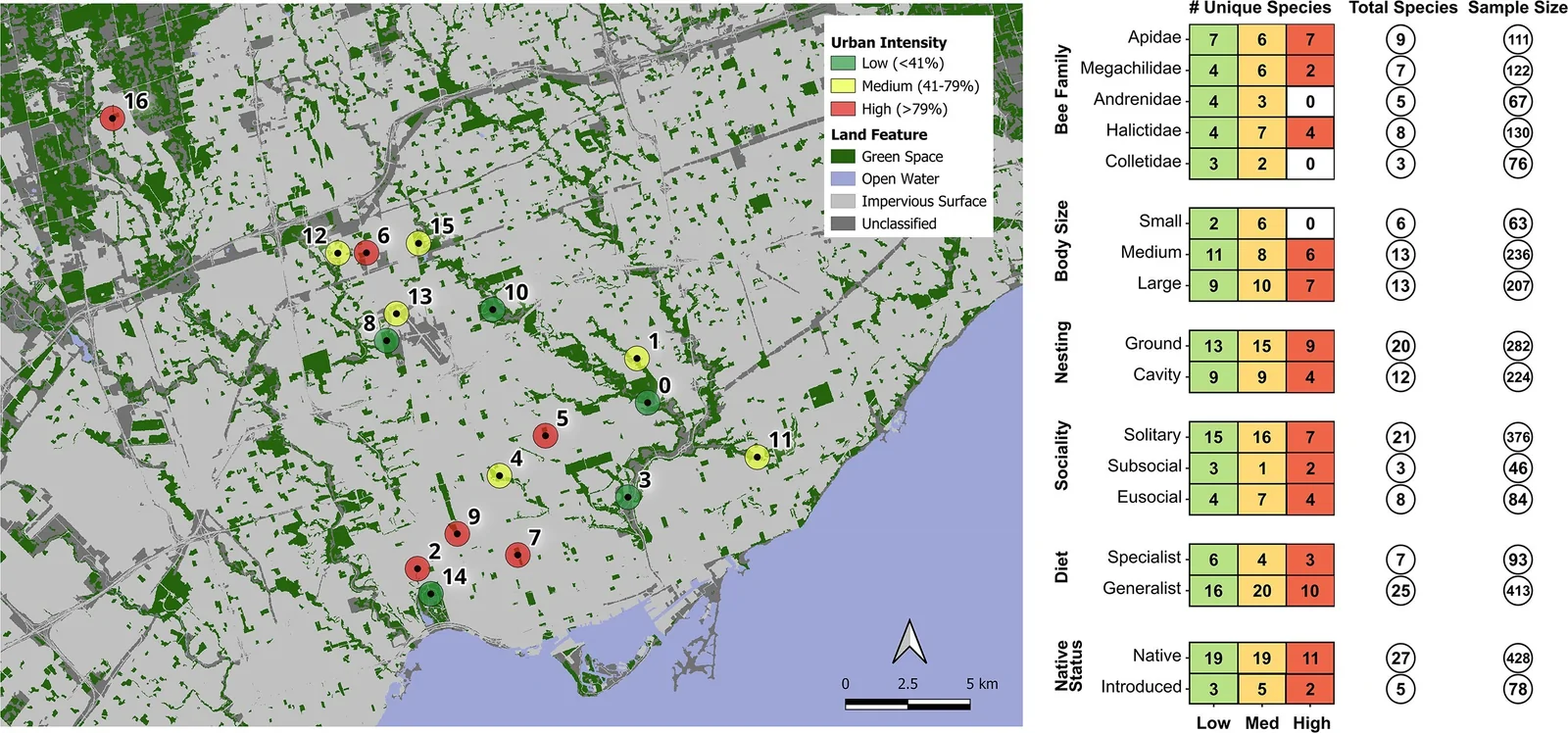
Sample sites across the Greater Toronto Area, Canada (*N* = 17). Outer buffer zones (500 m radius) around site points are coloured by the urbanization intensity level based on the Southern Ontario Land Resource Information System (SOLRIS) 3.0. Impervious surfaces include transportation and impermeable land; green space includes pervious surfaces, green spaces such as agricultural land, forest/tree/peat cover; unclassified includes undifferentiated land, rock, quarries, and land extractions. Tile plot next to map indicates which bee families/functional groups were detected per urban intensity; numbers inside tile indicate how many unique bee species for that category were found. Circles with numbers indicate total number of species for a bee family/functional group and sample size for each group. *N* = 32 bee species in total.

### DNA extraction and sequencing

We homogenized whole bee specimens, to ensure we captured all potential microbial and plant DNA that interact with the bee (internally and externally), using mortar and pestle and a homogenizer with ~0.1 ml of glass beads and one 1 mm steel bead. Extraction of DNA extraction followed the Qiagen DNeasy Blood & Tissue (catalogue number 69506) for purification of total DNA from whole-body animal tissue (DNeasy 96 protocol). DNA concentration was checked with a QuBit Fluorometer prior to sequencing. Library preparation, sequencing, and demultiplexing was done at the Canadian Centre for DNA Barcoding (Guelph, Canada). We included blank samples, which consisted of wells filled only with sterile water. We left the last well (H12) of our submitted plates empty as an additional control.

Paired-end amplicon sequencing was performed using Illumina MiSeq targeting 300 bp regions for three loci: the 16S rRNA gene (hereafter 16S) to identify bacteria, the internal transcribed spacer (hereafter ITS1) for fungi, and the RuBisCo large subunit gene (hereafter rbcL) to amplify plant DNA. We used the 16S forward primer 799F-mod3 (CMGGATTAGATACCCKGG) and the reverse primer 1115R (AGGGTTGCGCTCGTTG) to amplify the V5–V6 region for bacteria, the ITS1F forward primer (CTTGGTCATTTAGAGGAAGTAA) and ITS2 reverse primer (GCTGCGTTCTTCATCGATGC) to amplify the ITS1 region in fungi, and the rbcL7 forward primer (CTCCTGAMTAYGAAACCAAAGA) and rbcL8 reverse primer (GTAGCAGCGCCCTTTGTAAC) for rbcL7 fragment amplification. Samples were sequenced in triplicates, and all library preparation, sequencing, and demultiplexing of reads was completed by the Canadian Centre for DNA Barcoding (Guelph, Canada) and were provided as raw FASTQ reads for further analysis.

### Taxonomy classification and decontamination

We checked quality of the raw reads and trimmed them using the DADA2 pipeline (Callahan et al. 2016) in Qiime2 version 2023.9 (Bolyen et al. 2019). Reads were trimmed if Phred quality scores were below 40, and reads were truncated to keep lengths of 249 bp for 16S and 150 bp for rbcL. Due to variable lengths of ITS1 in fungi, we trimmed ITS1 adaptors using *cutadapt* version 4.9 (Martin 2011), following the DADA2 1.8 workflow for ITS sequences (https://benjjneb.github.io/dada2/ITS_workflow.html). We did not prefilter reads to remove sequences with ambiguous bases to maintain as many reads as possible and used the suggested minimum length of 50 bp to remove spurious low-length reads. In total, we obtained 9185 amplicon sequene variants (ASVs) for bacteria, 10 629 ASVs for fungi (read length range 52–474 bp, mean length of 220.52 bp), and 3214 ASVs for plants. Next, we obtained feature tables containing ASV sequences and abundances for each gene, with ASV sequences used for taxonomy classification via *blastn* version 2.14.1 (Altschul et al. 1990) and the core nucleotide *core_nt* database (last updated 23 September 2024). We kept the top 10 alignment hits for each gene using 10 maximum targeted sequences (–max_target_seqs 10). We then extracted the taxonomy identifiers (i.e. ‘taxids’) for all blast hits and processed them through Entrez E-utilities via *efetch* (version 15.3) to obtain full lineage information for each taxid (Sayers 2009). Simultaneously, the SILVA (version 138 full length sequences with 99% sequence similarity) database was used for taxonomy classification of bacterial ASVs (Quast et al. 2013, Yilmaz et al. 2014, Glöckner et al. 2017), the UNITE v9.0 database (version 18.07.2023 with global and 3% distance singletons) for fungal ASV classification (Abarenkov et al. 2023), and the nondereplicated rbcL database for plant ASV classification (Dubois et al. 2022). The majority of the hits for bacteria and fungi were labelled as ‘unclassified’ or ‘uncultured’ but had identical % identity and e-value scores to other taxonomy hits with full lineage information, so we filtered out these hits from our output. We only kept ‘unclassified’ and ‘uncultured’ hits if they at least maintained taxonomy identification up to family level. Next, to determine the most likely taxonomy up to genus level for each ASV, we used the remaining top alignment hits to identify the most common taxonomic classification. In cases where there were multiple likely classifications, the classification with the highest e-value score was used, followed by the highest % identity. If the e-value and % identities were identical for multiple top classifications, ASVs were classified up to the lowest common ancestor. Due to high purifying selection of the rbcL gene, discrimination of vascular plants is often poorer resulting in identical hits for many diverse plant species (Braukmann et al. 2017). Thus, we performed a filtering process by eliminating hits that identified a plant species, genus, or family which is not found in Ontario. To do this, we created a reference database by extracting all plant species (vascular and nonvascular) found in Ontario using the NatureServe Explorer database (3987 species records obtained) (NatureServe 2025). We also extracted all plant species found in Ontario from the Database of Vascular Plants of Canada (VASCAN; 3153 species records obtained) (Brouillet et al. 2010) to ensure we did not miss Ontario plants due to plant name synonyms which could occur. We finally used the Qiime2 databases to corroborate blast identifications or classify any ASVs that did not produce a blast hit or full taxonomic classification.

Next, we further filtered the bacterial, fungal, and plant datasets by entirely removing ASVs identified to be a true contaminant in blank samples using *phyloseq* (McMurdie and Holmes 2013) and *decontam* (Davis et al. 2017) packages in R version 4.2.1 (R Core Team 2021) using the prevalence method with a threshold of 0.1. If an ASV was significantly detected as a contaminant in a blank, that ASV was removed. Additionally, ASVs matching to domains not belonging to the gene were removed. For example, in bacteria we removed ASVs classified to Eukaryota. We also removed environmentally ubiquitous symbionts such as *Sodalis, Arsenophonus, Rickettsia, Spiroplasma*, and *Wolbachia* (Gerth et al. 2015, Graystock et al. 2017, Sless et al. 2025), and family classified to ‘Mitochondria’ or ‘Chloroplast’. For fungi, we removed ASVs classified to Bacteria or belonged to nonfungal phyla. For plants, we removed ASVs if they matched to phyla other than Streptophyta. After removal of blank samples and sample replicates with 0 reads, remaining sample replicates were merged together by sample and ASVs with abundances below a relative threshold abundance of 0.01% within a sample was converted to 0. Finally, we removed ASVs or samples with 0 total abundance, resulting in 6423 bacterial ASVs in 502 samples, 5694 fungal ASVs in 506 samples, and 2785 plant ASVs in 506 samples.

### Diversity analyses

For alpha and beta diversity analyses, we used both the regular ASV datasets and rarefied versions. We rarefied each sample to a minimum depth of 1000 reads using the *rarefy_even_depth* function from the *phyloseq* package. After rarefying, this resulted in 331 bacterial samples with 3620 ASVs, 506 fungal samples with 5694 ASVs, and 505 plant samples with 3309 ASVs. We compared diversity across bee species, bee genera, families, bee functional traits (bee body size, nesting behaviour, sociality, diet, and native status) and urbanization levels. First, to determine if taxa richness varied significantly across sample groups, we calculated alpha diversity using the Shannon–Wiener diversity metric in the *vegan* R package (Oksanen et al. 2020). Next, a Shapiro–Wilk test via the *shapiro.test* function checked for normality in alpha diversity. For nonparametric analyses, we used Kruskal–Wallis testing via *kruskal.test* followed by Dunn *post hoc* testing with *dunnTEST* from the *fsa* package with Bonferroni correction (Ogle et al. 2023) for significant Kruskal–Wallis tests. Otherwise, we performed parametric tests with ANOVA using the *aov* function followed by Tukey’s Honest Significance Difference tests using *HSD.test* from the R package *agricolae* (de Mendiburu 2023) for significant ANOVA results. To test if taxa composition varied significantly across sample groups, we calculated beta diversity using Bray–Curtis dissimilarity on the full ASV and rarefied datasets that we first transformed using the ‘Hellinger’ method from the *decostand* function within *vegan*, followed by creating a distance matrix using *vegdist* with method set to ‘bray’. Next, we used PERMANOVA using the *adonis2* method from *vegan* to check for significant differences in microbiome and plant composition for the different bee species, genera, families, traits, and urban intensities. This was followed by checks for beta dispersion using *anova* with *betadisper*; if ANOVA was significant, *post hoc* testing was done using Tukey HSD. To visualize Bray–Curtis dissimilarity, we created principal coordinate analysis plots by extracting the eigenvalues from weighted classical multidimensional scaling (*wcmdscale* function from *vegan*) to preserve Euclidean distances. Due to similar results between rarefied and non-rarefied datasets, the results and discussion will focus on results from the nonrarefied datasets.

Next, we merged ASVs with identical taxonomy classifications to assess differences in microbiome and plant composition at the taxon genus level. Prior to this, we removed ASVs not assigned to genus level to ensure focus on well classified taxa. For bacterial ASVs classified with the SILVA database, genera or families labelled with database codes (e.g. AD3) instead of being blank were treated as unique genus or family names to maintain as many unique taxonomy lineages as possible. To determine which taxa contributed the most to observed differences between groups, we implemented a similarity percentage (SIMPER) analysis using Bray–Curtis dissimilarity in the PAST program (Hammer et al. 2001). This analysis calculated a contribution value for each taxon to the overall dissimilarity between groups e.g. bee species, bee genus, bee family, bee functional traits, and urban intensity). To identify taxa shared across all analyzed groups or taxa associated to a particular group, we extracted bacteria, fungi, and plants using the genus merged datasets if they had ≥1.0% relative abundance and ≥50% sample prevalence within the sample type. We used the thresholds discussed in Graystock et al. (2017) to classify a core taxon for a sample type (e.g. samples belonging to a specific bee species).

### Taxa overrepresentation and co-expression

We incorporated random forest classification (RFC) to determine if bacterial, fungal, or plant compositions can predict bee species, bee genus, bee family, bee functional trait, or urban intensity. To do this, we used the R package *randomforest* (Liaw and Wiener 2002) for nine different training set sizes ranging from 10% to 90%, repeating the model 10 times to obtain an average accuracy. We used the genus merged datasets with unidentified genera removed and ran the *tuneRF* method to determine optimal ‘mtry’ values for each dataset—which calculates the minimum number of taxa to select to minimize error rates—for each dataset for 5000 runs (ntree = 5000). We used the *caret* package (Kuhn 2008) to generate a confusion matrix which summarized the RFC model performance, and we used the package *randomforestexplainer* (Paluszynska et al. 2020) to extract the top 10 important features (i.e. taxa) contributing to the RFC model accuracy. We focused on the top 10 taxa for all runs using the 80% training set size as using an 80/20 split for training/testing is standard practise and offers enough data for model training and predicting (Gholamy et al. 2018). Taxa that showed up all 10 times as an important feature were considered highly influential for the RFC model and likely play a role in taxa community differences for the analysed group. Finally, to assess if bees with specific functional traits experienced significant shifts in taxa alpha diversity with increasing urbanization, we implemented mixed-effects models. We used the *lmer* function from the *lme4* package (Bates et al. 2015), keeping sites as the random effect and urban intensity as an interacting factor with each functional trait. We included bee species, bee genus, and bee family as a fixed effect to account for natural microbial or plant association variation across bee taxonomic groups and repeated this model for each functional trait individually to avoid multicollinearity.

## Results

### Sequencing and sample output

A total of 506 bees were collected from low (222 individuals), medium (226), and high (58) urban intensities. This included bees from five families encompassing 16 genera and 32 species, with the most specimens belonging to Halictidae (130 individuals; 8 species), then Megachilidae (122; 7), Apidae (111; 9), Colletidae (76; 3), and the least from Andrenidae (67; 5). All bee species were sampled at all levels of urban intensity except for andrenid or colletid individuals which were not found in high urban intensities (Fig. 1, Tables S1 and S2). All bee functional groups were collected at each urban intensity except small bee species, which were absent from high urban intensities (Fig. 1, Tables S1 and S2).

Amplicon sequencing resulted in a total of 35 526 785 reads for 16S (average 20 560 reads/sample replicate), 33 900 993 reads for ITS1 (average 19 630 reads/sample replicate), and 61 503 775 reads for rbcL (average 35 613 reads/sample replicate). Average read count of ASVs after filtering and using a 0.01% relative abundance threshold was 5555 reads/sample replicate for bacteria (8 300 320 reads; Table S3), 19 111 reads/sample replicate for fungi (28 991 482 read; Table S4), and 31 966 reads/sample replicate for plants (48 519 958 reads; Table S5). Merging of ASVs by genus resulted in 598 bacterial genera (292 families + 48 unclassified families), 830 fungal genera (308families + 33 unclassified families), and 252 plant genera (89 families + 5 unclassified families) (Tables S3, S4, and S5, respectively).

### Microbiome and plant diversity across bee taxonomic groups and traits

Across all bee taxonomic levels, bacterial alpha diversity significantly differed across all levels whereas fungal alpha diversity only differed at the bee species level (Table S6). At the family level, Apidae and Andrenidae had significantly richer bacterial microbiomes compared to Halictidae (Kruskal–Wallis ꭓ^2^ = 31.2, df = 4, *P* < .001; Fig. 2a, Table S6). However, bacterial richness varied greatly within families at the genus level, where instead the megachilid genus *Heriades* showed the highest bacterial richness, which was significantly greater when compared to the other megachilid genera *Megachile, Anthidium*, and *Pseudoanthidium* (Fig. S1a, Table S6). Even within genera there was high variation, with *Heriades leavitti* exhibiting the highest bacterial richness (mean Shannon index = 2.7) whereas *H. carinata* had the lowest (mean Shannon index = 0.06) (Table S6). Fungal diversity was similar across bee families (Fig. 2b, Table S7) and bee genera (Fig. S1b, Table S7) but showed variation in species even within the same genus (Fig. S2b, Table S7). For example, *Andrena helianthi* had significantly greater fungal diversity compared to all other *Andrena* species (Table S6). There were significant differences in bacterial and fungal diversity at all bee taxonomic levels, however, composition was largely similar across bee groups (Fig. 2b, Figs S1b and S2b, Table S7). Differences in microbial communities were attributed to within-group variation of taxon compositions (Fig. S4a and b, Table S8).

**Figure 2:**
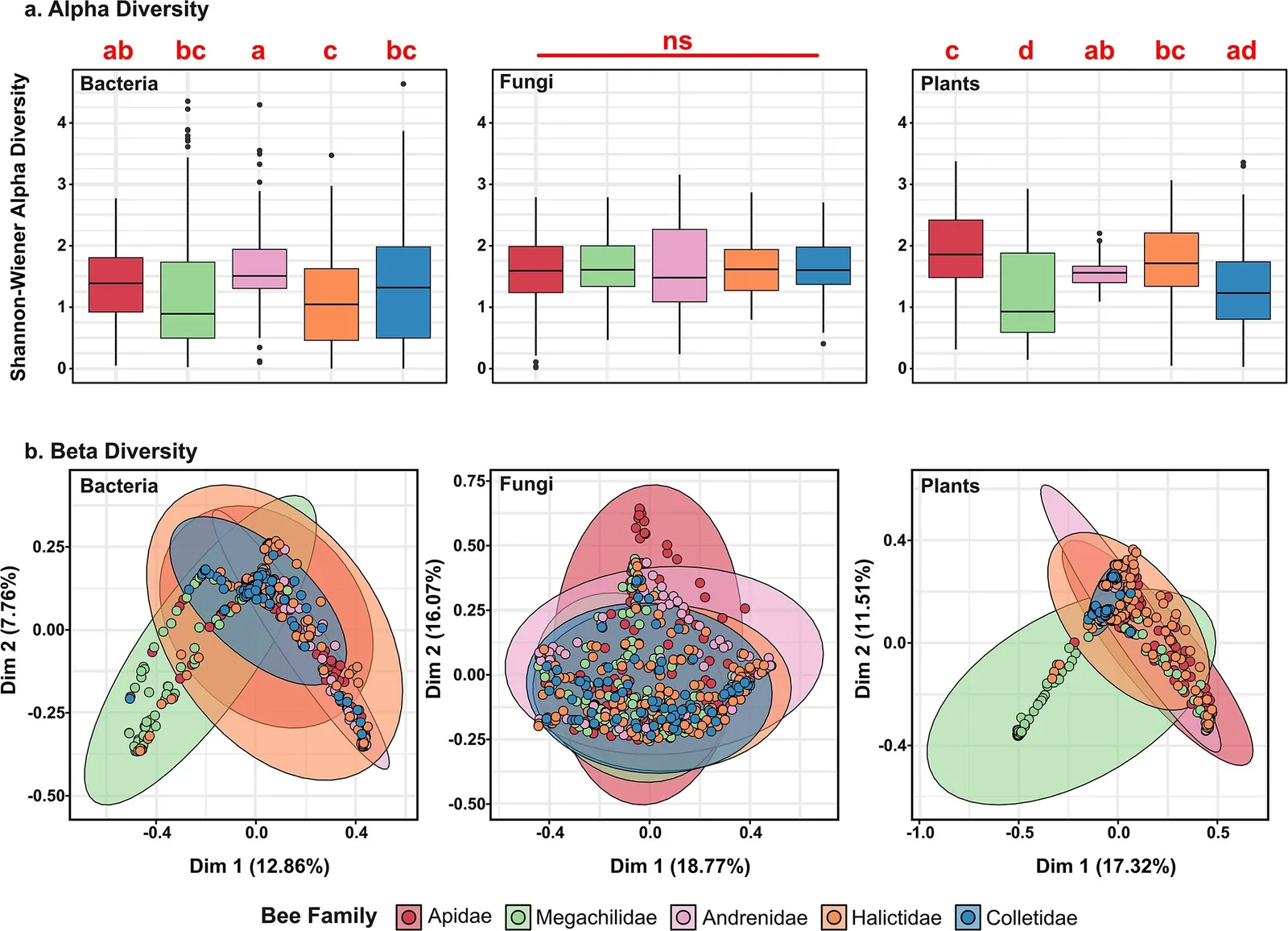
Diversity plots of unrarefied data by bee family for bacteria (16S rRNA), fungi (ITS1), and plants (rbcL) using (a) Shannon–Wiener alpha diversity metrics and (b) weighted classical multidimensional scaling (*wcmdscale*) for beta diversity. Red letters above boxplots represent significantly different families; ns = no significance.

Examination of bees by functional trait resulted in significantly higher bacterial alpha diversity higher in subsocial and native bees when compared to eusocial and introduced species (Fig. S3a, Table S6). Fungal diversity was instead found to be significantly higher in large bee species and solitary species when compared to medium-sized species and subsocial species (Fig. S3a, Table S6). Beta diversity of bacteria and fungi was significant across all examined functional traits, but clustering was only evident for a portion of solitary bees for bacteria (Fig. S3b).

Plant diversity differed at all bee taxonomic levels, with plant diversity significantly richer in Apidae compared to Andrenidae, Colletidae, and Megachilidae (Kruskal–Wallis ꭓ^2^ = 78.1, df = 4, *P* < .001; Fig. 2a, Table S6). The halictid species *Augochlorella aurata* had the highest plant diversity (mean Shannon index = 2.7) whereas the colletid *Hylaeus modestus* had the lowest (mean Shannon index = 0.58; Table S6). Plant beta diversity significantly differed across all bee taxonomic groups (Table S7), with a portion of Megachilidae forming a cluster (Fig. 2b), which consisted of *Megachile rotundata* and *Anthidium oblongatum* (Figs S1b and S2b). Colletidae species and Andrenidae species also formed distinct clusters due to highly diverse plant associations (Fig. S3b). For instance, a portion of Andrenidae were highly associated with the plant genera *Xanthium* and *Tragopogon*, whereas Colletidae specimens were dominated by either *Frangula* or *Daucus*. Halictidae and Apidae were quite varied in their plant associations, whereas a large majority of Megachilidae were associated with *Lotus* (Fig. S4c). By bee functional trait, plant diversity significantly differed in all groups except for diet, where there were no differences in plant richness between specialists and generalists (Fig. S3a, Table S6). Instead, plant richness was significantly higher in large and medium-sized bees, ground nesters, subsocial and eusocial bees, and native bees compared to small bee species, cavity-nesters, solitary bees, and introduced species. Plant composition within bees significantly differed across all examined groups with pronounced clustering between native and introduced bees (Fig. S3b, Table S7).

Across all bee samples, we identified 13 common (i.e. ≥1% abundance and ≥50% prevalence across the sample group) microbes and plant associates; two bacteria, five fungi, and six plants (Fig. S5). Bacteria *Apilactobacillus* and *Acinetobacter* occurred in 72% and 62% of all bee samples respectively and made up 31% and 15% of all bacterial reads (Table S9). Together these two bacteria contributed over 41% of the variation observed across all bee taxonomic levels and functional traits (Table S8) and were consistently identified as important taxa in our RFC models when predicting bee family, bee genus, or bee species (Table S10). The fungi *Cladosporium, Alternaria, Metschnikowia, Ascosphaera*, and *Epicoccum* were common in our bees, but only *Cladosporium* and *Alternaria* were 100% prevalent in all bee samples (Table S9). These two fungi also contributed over 50% of the community variation observed across most bee groups, and both fungi were always identified as an important taxon in distinguishing between bee taxonomic groups (Table S10). In the case of sociality, *Cladosporium* and *Hemimycena* contributed to 50% of the community composition variation between social groups (Table S8). The plant genera *Trifolium, Erigeron, Daucus, Cirsium, Lotus*, and *Medicago* were common across all bees, with *Trifolium* as the most prevalent (75%) and abundant (14%) (Table S9). Overall, due to the more diverse compositions of plant genera across bee families and species (Fig. S4c), plant diversity generally better predicted bee taxonomy compared to bacteria and fungi in RFCs (Fig. 3, Table S10). For example, using an 80% training data set size plants had an average accuracy of 82%, 71%, and 69% in predicting bee family, bee genus, and bee species, respectively, which was higher than bacteria (58%, 53%, and 47%) and fungi (69%, 62%, and 60%). Plant diversity also better categorized bees based on functional traits, except for sociality where it was comparable where it had similar average accuracy (82%) to fungi (84%) and bacteria (83%) (Fig. 3, Table S10).

**Figure 3:**
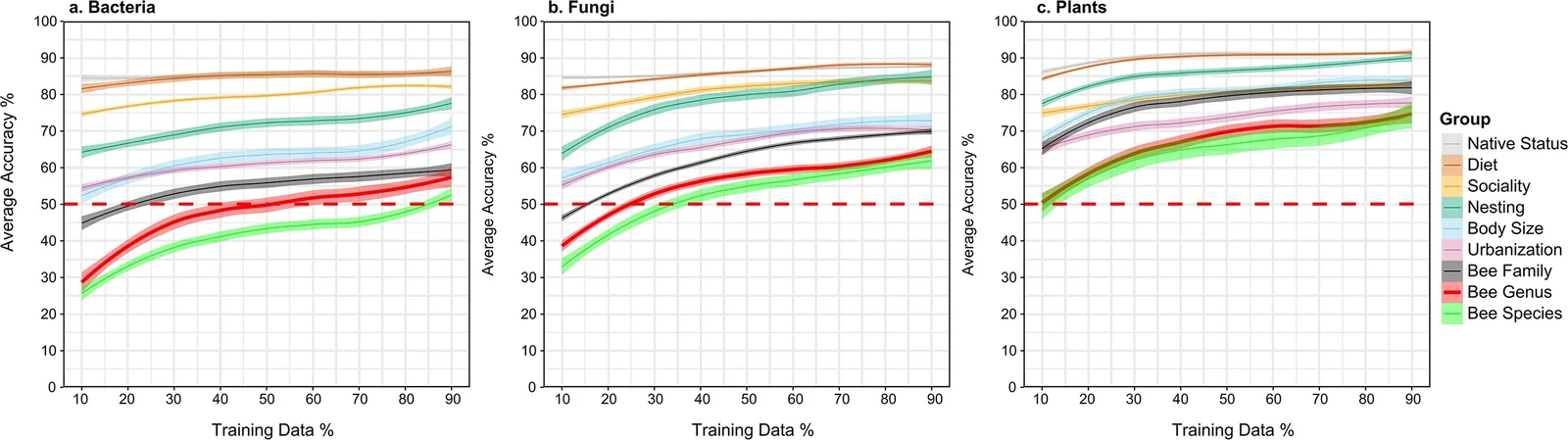
Average accuracy for each RFC model for (a) bacteria, (b) fungi, and (c) plants. Each RFC tested used genus merged relative abundance datasets to predict different bee groupings and urban intensity. Red dashed line indicates 50% average accuracy performance. Error bars created using loess smoothing. Full results in Table S10.

Across all taxonomic levels and functional traits, we found several microbes and plant associations that were predominantly associated to a specific bee species; no taxa were found common for a single bee family, bee genus, or functional trait (Fig. 4). For example, the bacteria *Commensalibacter* and *Gluconobacter* were most prevalent in *A. helianthi, Lactobacillus*, and *Fructobacillus* were primarily detected in *Bombus impatiens* and *Bifidobacterium* was most prevalent in *Bombus griseocollis* (Table S9). For fungi, focusing on species with more than two individuals, *Andrena carataegi* and *A. wilkella* associated with the fungi *Sawadaea* and *Cystofilobasidium*, respectively. Among the many common plant genera, several genera were common primarily to Apidae species, such as *Impatiens* and *Securigera* found primarily in *B. impatiens* or *Silphium* and *Centaurea* primarily found in *Melissodes trinodis*.

**Figure 4:**
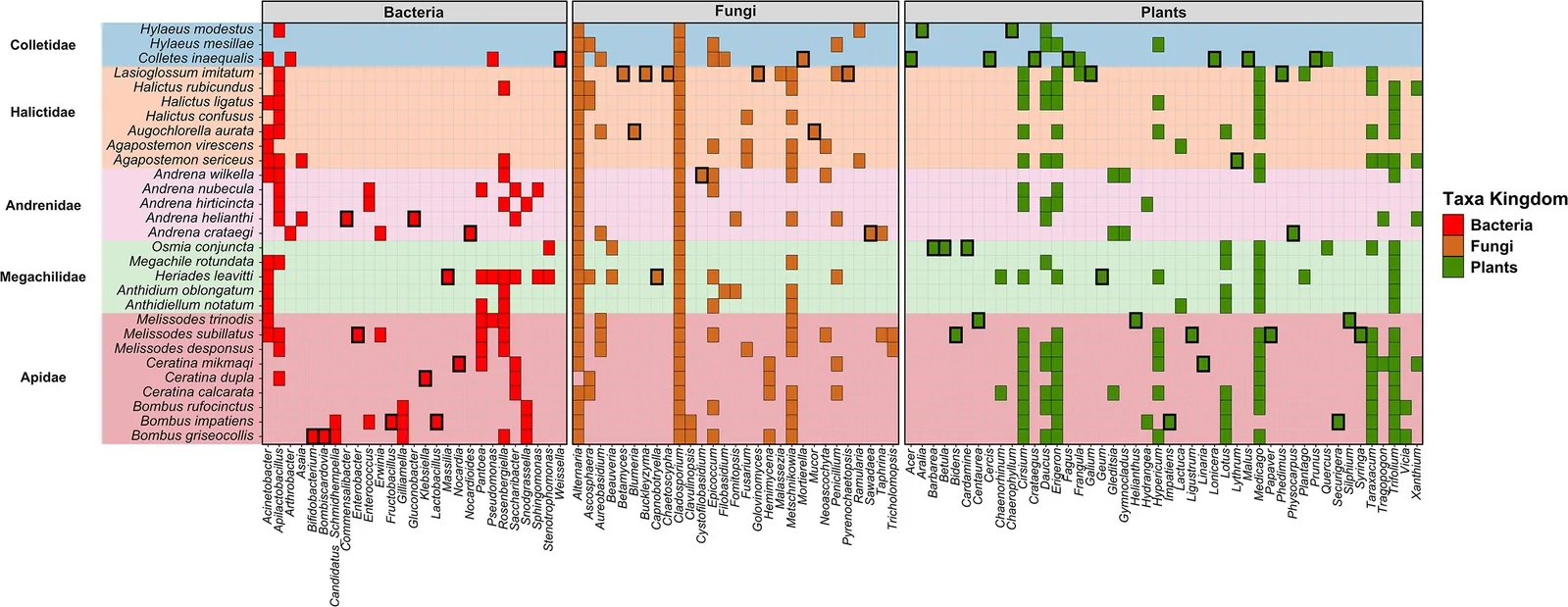
Common microbes and plant associations found across bee species with a total relative abundance ≥1% and prevalence ≥50% across the species. Microbes and plant genera highlighted with a black box indicate a unique association of that taxon to a single bee species only.

### Urbanization impacts on microbiome and plant associations

Overall, bees sampled along an urban gradient did not significantly differ in fungal richness, but bacterial richness was higher in bees sampled from low urban intensities compared to high intensities (Kruskal–Wallis ꭓ^2^ = 8.6, df = 2, *P* = .014, Fig. 5a, Table S6). Bacterial and fungal composition significantly differed across urban intensities and at all bee taxonomic levels primarily due to within-group variation, as clustering was not evident (Fig. 5b, Table S7). For bee species with representation across the urban gradient, we found that urbanization did not impact bacterial diversity but fungal richness were found to be elevated in *Halictus rubicundus* individuals from medium intensities relative to high intensities, and in *M. rotundata* from medium and high urban intensities relative to low urban intensities (Table S11). We found that the bacterium *Rosenbergiella* and fungus *Filobasidium* was common in bees only from high urban intensities, whereas the bacterium *Saccharibacter* and fungi *Ascosphaera* and *Epicoccum* were common to bees from low intensities; bees from medium urban intensities were instead associated with microbiota that were common across the urban landscape (Table S9). Furthermore we found no significant interaction between urban intensity and any of the bee functional traits for bacterial and fungal richness (Table S11).

**Figure 5:**
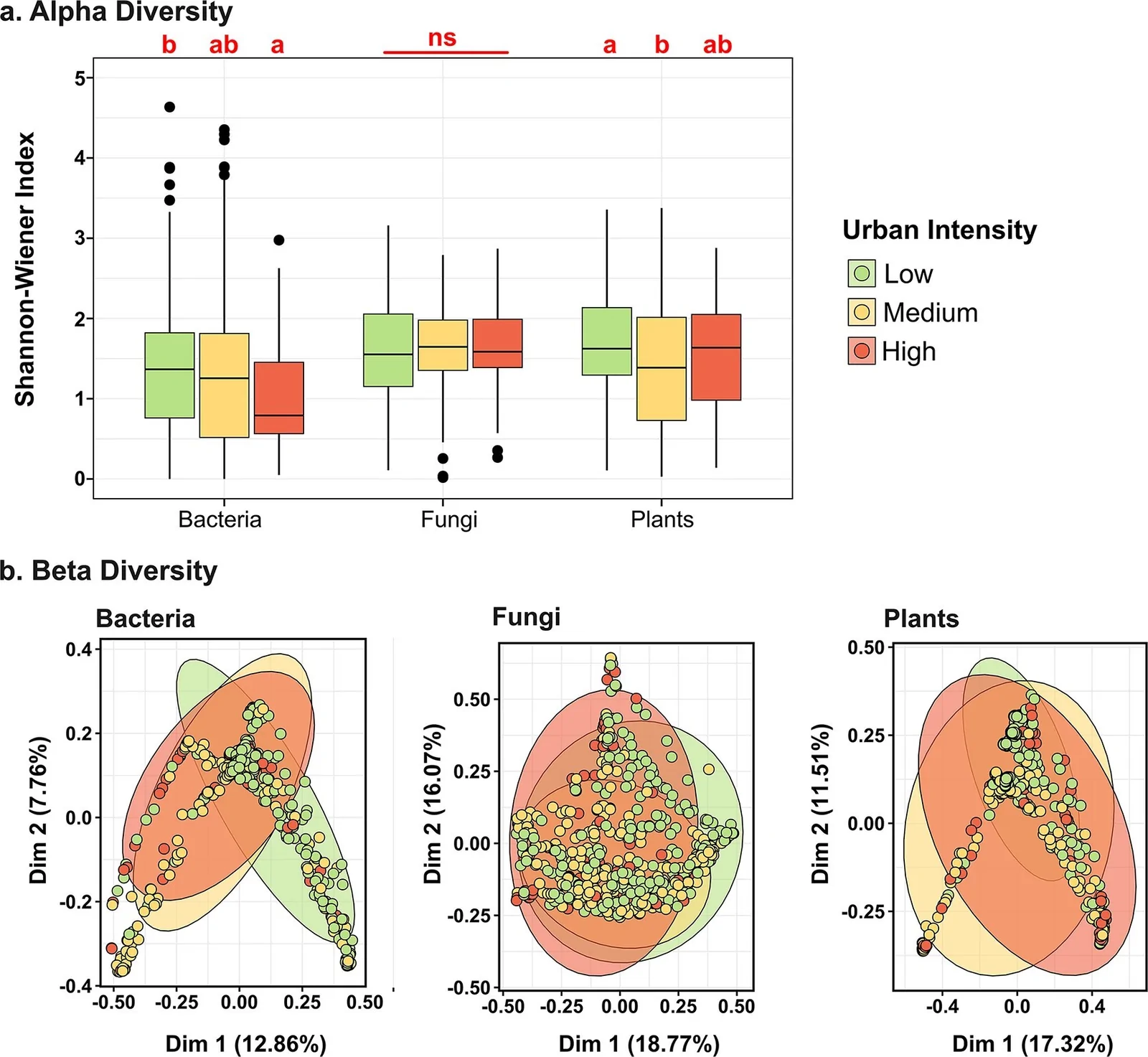
Diversity plots of unrarefied data by urban intensity for bacteria (16S rRNA), fungi (ITS1), and plants (rbcL) using (a) Shannon–Wiener alpha diversity metrics and (b) weighted classical multidimensional scaling (*wcmdscale*) for beta diversity. Red letters above boxplots represent significantly different families; ns = no significance.

With regards to plant associations, bees from low urban intensities had significantly higher plant diversity compared to bees from medium urban intensities (Kruskal–Wallis ꭓ^2^ = 18.0, df = 2, *P* < .001; Fig. 5a, Table S6) but composition was largely similar across the urban gradient (Fig. 5b). Conversely, plant diversity in bee species represented across the urban gradient showed no significant differences (Table S12). Regardless, plant composition still performed better at predicting the urban intensity the bees were collected from (average accuracy = 77%) compared to bacteria (64%) or fungi (71%) (Fig. 3, Table S10). Our mixed-effects models distinguished a single significant interaction with urbanization and native status, where introduced bees from high urban intensities showed increased plant richness compared to native bees from low urban intensities (estimate = 0.5, standard error = 0.22, df = 281.2, *P* = .029; Table S11). We found several plant genera common in bees from specific urban intensities, such as *Lotus* common in bees from high urban intensities, *Hypericum* to medium urban intensity bees, and *Xanthium, Frangula, Cirsium*, and *Taraxacum* for low urban intensity bees (Table S9).

## Discussion

This study examines the microbiome and plant associations for 32 bee species from five families (Apidae, Megachilidae, Colletidae, Andrenidae, and Halictidae) across different bee taxonomic levels, functional traits, and urbanization. We found differences in bacterial and plant diversity at all bee taxonomic levels, whereas fungal diversity was different between bee species. Bacterial diversity was highest in subsocial bees and native bees, whereas fungal diversity was higher in large species and solitary species. All functional traits except diet impacted plant diversity, where bee-associated plant diversity was highest in large species, ground-nesters, subsocial species, and native species. We identified two bacteria, five fungi, and six bee-associated plant taxa that were highly common across the bee community, as well as several distinct bee-taxon associations for certain bee species. Bees from low urban intensities had increased bacterial richness when compared against bees collected from highly urbanized areas. Increasing urbanization had little impact on fungal richness for most wild bees. The impacts of urbanization were most notable for bee-plant associations with both increased and decreased plant richness at higher urban levels depending on functional trait. Plant composition data was also better at predicting bee taxonomic level or functional trait than microbial data but was comparable when predicting sociality.

### Distinct microbial and plant associations with bee species

Five bee families shared many common bacterial and fungal microbes, most of which are well known core taxa in bees such as the bacteria *Acinetobacter* and *Apilactobacillus* (Graystock et al. 2017, Caesar et al. 2024, Nguyen et al. 2024), fungi *Cladosporium* and *Alternaria* (Nguyen and Rehan 2023, Rutkowski et al. 2023), and plant resources such as *Trifolium, Erigeron*, and *Taraxacum* (Nguyen et al. 2024). However, there were several microbial and plant taxa associations to specific bee species which suggests important microbial influences on specific bee species.

Although our study focuses on environmental symbionts across the whole bee specimen whereas most microbiome studies focus solely on the gut, we found well-known bee symbionts based on previous gut microbiome studies to also be common in our bee species (Kwong and Moran 2016, Graystock et al. 2017, Motta and Moran 2024). For instance, *Commensalibacter*, a noncore, environmentally acquired acetic-acid symbiont commonly found in the guts of insects that specialize on resources high in carbohydrates (i.e. Apidae species such as honey bees, bumble bees, and small carpenter bees) (Botero et al. 2023), was mainly common in *A. helianthi* (a nonapid bee) but was not highly prevalent in the Apidae species in our study (e.g. *Bombus* spp. and *Ceratina* spp.). *Andrena helianthi*, an Asteraceae specialist, associated with three plant genera two of which are asters (i.e. *Xanthium* and *Tragopogon*) whereas *Bombus* and *Ceratina* in our study associated with several more Asteraceae genera (Table S9). However, we speculate the high abundance and prevalence of *Commensalibacter* in *A. helianthi* may be less a result of diet and more to do with immunity. One study found that *Commensalibacter* suppressed the spread of *Gluconobacter morbifer*, a pathogenic sugar-loving environmental bacterium, in *Drosophila* through competition (Crotti et al. 2010). *Commensalibacter intestini*’s relative abundance also increased in the guts of honey bees infested with the mite *Varroa*, further suggesting a role in immune defense for bees (Hubert et al. 2017). In our study, *Gluconobacter* was also identified as a common endosymbiont in *A. helianthi*, with identical prevalence as *Commensalibacter* (69% prevalence for both) but a lower abundance (*Gluconobacter*: 1.1%, *Commensalibacter*: 2.6%) (Table S9). It is possible that *Commensalibacter* may be playing an important role in immunity for *A. helianthi* by outcompeting with *Gluconobacter*, however further testing of these specific microbes in *A. helianthi* is warranted to confirm if this is the case. Interestingly, we identified *Bifidobacterium* with at least 50% prevalence and 1% abundance primarily in *B. griseocollis* and not the other *Bombus* species in our study. Instead, it had 0% prevalence in *B. rufocinctus* but 80% prevalence in *B. impatiens* (<1% relative abundance in both species) in our study. *Bifidobacterium* is considered a core honey bee and bumble bee microbe along with other symbionts such as *Gilliamella, Lactobacillus*, and *Snodgrassella*, all of which were also prevalent in all our *Bombus* species, except for *Lactobacillus* which was mainly detected in in *B. impatiens. Bifidobacterium* is vertically transmitted through the interactions of colony members, and its varied prevalence may be the result of natural variation in the transmission of this bacterium within different colonies, as observed in other studies where *Bifidobacterium* is present in most *Bombus* species but not all of them (Kwong et al. 2017, Zhang et al. 2021). Another aspect that impacts the variation of core phylotypes is the presence of noncore microbes which can displace core symbionts especially in wild-caught bumble bees with greater, natural exposure to noncore microbes (Hotchkiss et al. 2025). However, future focused research examining microbial functions in different wild bee species will be necessary to elucidate the direct biological and functional roles of microbes across diverse bee taxa.

Meanwhile, there were fewer unique associations of specific bee species with fungi due in part to the largely similar fungal microbiome across wild bees (Fig. 2). Unlike bacterial symbionts that show strong host associations, fungal communities are shaped by local environment which have previously been shown to be resilient towards urbanization (Hui et al. 2017, Abrego et al. 2020). The fungal communities in wild bees likely represent transient environmental associates rather than obligate bee symbionts. Within this context, two Andrenidae species, *Andrena crataegi* and *A. wilkella* had unique associations with the fungi *Sawadaea* and *Cystofilobasidium*, respectively. *Sawadaea* is a powdery mildew fungus and an obligate parasite of maple (*Acer*) trees (Hirose et al. 2005). *Acer* trees were not common in *A. crataegi*, nor were there any ASVs assigned to *Acer* present in any *A. crataegi* sample. However, other trees such as *Gleditsia* and *Gymnocladus* were common in *A. crataegi* but there are no studies to suggest that *Sawadaea* can exist on nonmaple woody trees. Our result may reflect the specific site of *A. crataegi* samples, which was only from Site 4, a medium urban intensity site. We speculate that while *Acer* is not a main floral resource for *A. crataegi, Acer* trees infected with *Sawadaea* in the area could come into contact with *A. crataegi* individuals that nest in the area from wind-borne conidia (Qin et al. 2023). *Cystofilobasidium* is a yeast that partakes in breakdown of polysaccharides like pectin, which is present in pollen grain walls (Ye et al. 2021). Little is known in terms of this fungus’ relationship with solitary bees, but its association only with *A. wilkella* suggests an increased nutrient uptake from pectin-rich pollen grains which may be the type of pollen it encounters in its floral preferences, all of which are Fabaceae genera. Further investigation into the pollen structure of its legume-associated floral resources is needed to determine if the grain walls are rich in pectin, which would support the idea that *Cystofilobasidium* partakes in its breakdown.

There were many distinct plant associations with a single bee species, which were mainly from generalist bee species. Our findings show that diet specialization played no role in altering the microbiome, nor did it produce any significant difference in plant associations between specialists and generalists (Fig. S3). We attribute this lack of difference in plant diversity between the diet types because of floral constancy—the preference of foraging on one flower—exhibited by generalist bees (Kelly and Elle 2021, Grüter et al. 2024). Rather, our distinct bee–plant associations may be attributed to other factors such as nesting behaviour. For instance, *Ceratina mikmaqi*, a stem-nesting bee, associated with *Linaria*, a woody plant that can offer pithy stem material for nesting (Mikát et al. 2016). *Bombus impatiens* associated with, unsurprisingly, *Impatiens* emphasizing its floral preference (and namesake) despite being capable of a generalist diet. Nevertheless, two specialist *Melissodes* species in our study did have distinct plant associations. *Melissodes subillatus* is an Asteraceae specialist (Gibbs et al. 2023) but did associate with *Syringa* and *Ligustrum* plant genera belonging to the Oleaceae plant family suggesting that this specialist has flexibility in floral choice, which sometimes occurs in specialists when other resources are present and abundant (Burger et al. 2021). *Melissodes trinodis* is also an Asteraceae specialist (Gibbs et al. 2023) and its associations with *Helianthus, Silphium*, and *Centaurea* (Asteraceae genera) corroborate its diet specialization.

### Urbanization impacts bee–plant and bee–bacteria associations more so than bee–fungi interactions

While fungal alpha diversity across bee species was unaffected by urban intensity, bacterial diversity was higher in bees from low urban intensities compared to high intensities. This finding generally agrees with previous studies that show bee bacterial and fungal communities change with urbanization (Nguyen and Rehan 2022, 2023, Chau et al. 2023, Peters et al. 2025). Although, focusing on five species with representation across the urban gradient revealed that urbanization did not impact bacterial communities at the species level, but rather, increasing urbanization impacts the bacterial community at the bee community level or only for some bacteria. For example, one study found that urbanization significantly decreased the bacteria *Snodgrassella* and *Nocardioides* in solitary bees whereas other bacteria were unaffected (Fernandez De Landa et al. 2023). As such, bee community-level shifts in bacterial communities are nuanced but reveal complex high-level patterns that are important for broader ecological impacts on urban bee communities. These changes may represent novel challenges for wild bees because host-associated bacteria contribute to nutrient acquisition, digestion, immune defense, and overall host fitness (Engel et al. 2016, Kwong and Moran 2016, Dharampal et al. 2020). On the contrary, it could be that our five representative bees are robust species that are unaffected by urbanization whereas other bee species might be influenced more readily by urbanization.

Instead, urbanization had a profound impact on plant diversity. Bees from low urban intensities had high plant richness, which was similar to bees collected from high urban areas. Furthermore, plant beta diversity varied significantly across the urban landscape, but clustering of plant communities by urban intensity was not evident. This suggests that while low urbanization may allow bees to access more floral resources, available plant species across the urban landscape appear homogenous. This may be attributed to pollinator programs where cities such as Toronto partake in initiatives to increase floral diversity in parks and urban gardens to boost pollinators richness, which may be one reason we observe similar plant-associated compositions in across the bee community (MacIvor 2015). Indeed, when assessing changes in plant richness in bee species with individuals sampled along the urban gradient, we observed no significant differences likely due to similar resources present at all urban intensities. While plant diversity was impacted by differences in functional traits, the impacts of urbanization were negligible when accounting for trait-based variation. This finding indicates that while bees from different functional guilds show floral preferences, increasing urbanization does not appear to hinder their floral choice.

## Conclusion

This study highlights the diverse relationships between bee families and their microbial and plant associations. We showed that integrating a trait-based approach, urbanization, and phylogeny can reveal many unique interactions both at a bee community level and at finer resolutions. Bacterial diversity was higher in Andrenidae and Apidae relative to other families, and also higher in subsocial and native bees. Fungal diversity remained consistent across all bee families but was higher in large-bodied species and solitary species. Bee-associated plant diversity was higher in Apidae and Halictidae, and in large-bodied species, ground-nesters, subsocial and eusocial species, and native bees. Diet had no effect on microbial diversity or plant diversity in this study. Common microbes and plant-associations were already well-known core bee microbes and common plants in the sample region, but we also detected unique taxon associations for several bee species providing early indications of potential host-specific interactions that warrant further investigation. Overall, increasing urbanization greatly impacted plant diversity across focal bees, with little to no impact on microbial diversity suggesting that, despite common plants throughout the sample region, the effect of urbanization alters bee foraging behaviours and that higher urban intensities may be beneficial to certain bee groups via increased plant richness.

## Supplementary Material

fiag109_Supplemental_Files

## Data Availability

Sequence data can be found through NCBI under BioProjects PRJNA1124100 and PRJNA1242897. Data used for statistical analyses for bacteria, fungi, and plants have been included as in Supplementary_Data_File_1 and are found in supplementary tables S3, S4, and S5, respectively.

## References

[bib1] Abarenkov K, Zirk A, Piirmann T. et al. UNITE QIIME release for Fungi 2. Version 18.07.2023. UNITE Community, 2023. 10.15156/BIO/2938080.

[bib2] Abrego N, Crosier B, Somervuo P. et al. Fungal communities decline with urbanization—more in air than in soil. ISME J. 2020;14:2806–15. 10.1038/s41396-020-0732-1. PMC7784924 32759974

[bib3] Altschul SF, Gish W, Miller W. et al. Basic local alignment search tool. J Mol Biol. 1990;215:403–10. 10.1016/S0022-2836(05)80360-2. 2231712

[bib4] Anderson SE, Hahn P, Gonzalez G. et al. Land-use change alters specialist bee diet and drives body size declines. Landsc Ecol. 2024;39:115. 10.1007/s10980-024-01893-1.

[bib5] Ascher JS, Pickering J. Discover Life bee species guide and world checklist (Hymenoptera: Apoidea: Anthophila). Bugguide, 2024. http://www.discoverlife.org/mp/20q?guide=Apoidea_species (1 june 2025, date last accessed).

[bib6] Ayers AC, Rehan SM. Supporting bees in cities: how bees are influenced by local and landscape features. Insects. 2021;12:128. 10.3390/insects12020128. PMC7912800 33540577

[bib7] Ayers AC, Rehan SM. Bee-plant interaction and community response along an urbanization gradient. J Urban Ecol. 2023;9:juad006. 10.1093/jue/juad006.

[bib8] Bates D, Mächler M, Bolker B. et al. Fitting linear mixed-effects models using lme4. J Stat Softw. 2015;67:1–48. 10.18637/jss.v067.i01

[bib9] Bogati KA, Sewerniak P, Walczak M. Unraveling the effect of soil moisture on microbial diversity and enzymatic activity in agricultural soils. Microorganisms. 2025;13:1245. 10.3390/microorganisms13061245. PMC12195373 40572134

[bib10] Bolyen E, Rideout JR, Dillon MR. et al. Reproducible, interactive, scalable and extensible microbiome data science using QIIME 2. Nat Biotechnol. 2019;37:848–857. 10.1038/s41587-019-02099. PMC7015180 31341288

[bib11] Botero J, Sombolestani AS, Cnockaert M. et al. A phylogenomic and comparative genomic analysis of *Commensalibacter*, a versatile insect symbiont. Anim Microbiome. 2023;5:25. 10.1186/s42523-023-00248-6. PMC10149009 37120592

[bib12] Brasil SNR, George M, Rehan SM. Functional traits of wild bees in response to urbanization. J Insect Conserv. 2024;28:127–39. 10.1007/s10841-023-00528-1.

[bib13] Braukmann TWA, Kuzmina ML, Sills J. et al. Testing the efficacy of DNA barcodes for identifying the vascular plants of Canada. PLoS One. 2017;12:e0169515. 10.1371/journal.pone.0169515. PMC5224991 28072819

[bib14] Brouillet L, Coursol F, Meades SJ et al. VASCAN, the database of vascular plants of Canada. VASCAN, 2010. http://data.canadensys.net/vascan/ (7 October 2025, date last accessed).

[bib15] Burger H, Joos N, Ayasse M. Floral cues of non-host plants attract oligoletic *Chelostoma rapunculi* bees. Front Ecol Evol. 2021;9. 10.3389/fevo.2021.682960.

[bib16] Caesar L, Rice DW, McAfee A. et al. Metagenomic analysis of the honey bee queen microbiome reveals low bacterial diversity and Caudoviricetes phages. mSystems. 2024;9:e0118223. 10.1128/msystems.01182-23. PMC10878037 38259099

[bib17] Callahan BJ, McMurdie PJ, Rosen MJ. et al. DADA2: high-resolution sample inference from Illumina amplicon data. Nat Methods. 2016;13:581. 10.1038/nmeth.3869. PMC4927377 27214047

[bib18] Cardinal S, Buchmann SL, Russell AL. The evolution of floral sonication, a pollen foraging behavior used by bees (Anthophila). Evolution. 2018;72:590–600. 10.1111/evo.13446. PMC5873439 29392714

[bib19] Chau KD, Samad-zada F, Kelemen EP. et al. Integrative population genetics and metagenomics reveals urbanization increases pathogen loads and decreases connectivity in a wild bee. Global Change Biol. 2023;29:4193–211. 10.1111/gcb.16757. 37173859

[bib20] Crotti E, Rizzi A, Chouaia B. et al. Acetic acid bacteria, newly emerging symbionts of insects. Appl Environ Microbiol. 2010;76:6963–70. 10.1128/AEM.01336-10. PMC2976266 20851977

[bib21] Davis NM, Proctor D, Holmes SP. et al. Simple statistical identification and removal of contaminant sequences in marker-gene and metagenomics data. Microbiome. 2017:221499. 10.1101/221499 PMC6298009 30558668

[bib22] de Mendiburu F. agricolae: statistical procedures for agricultural research. R package version 1.3-7. CRAN, 2023. https://CRAN.R-project.org/package=agricolae.

[bib23] de Sousa LP. Bacterial communities of indoor surface of stingless bee nests. PLoS One. 2021;16:e0252933. 10.1371/journal.pone.0252933. PMC8270128 34242231

[bib24] Dew RM, McFrederick QS, Rehan SM. Diverse diets with consistent core microbiome in wild bee pollen provisions. Insects. 2020;11:499. 10.3390/insects11080499. PMC7469187 32759653

[bib25] Dharampal PS, Hetherington MC, Steffan SA. Microbes make the meal: oligolectic bees require microbes within their host pollen to thrive. Ecol Entomol. 2020;45:1418–27. 10.1111/een.12926.

[bib26] Dubois B, Debode F, Hautier L. et al. a detailed workflow to develop QIIME2-formatted reference databases for taxonomic analysis of DNA metabarcoding data. BMC Genom Data. 2022;23:53. 10.1186/s12863-022-01067-5. PMC9264521 35804326

[bib27] Engel P, Kwong WK, McFrederick Q. et al. The bee microbiome: impact on bee health and model for evolution and ecology of host-microbe interactions. mBio. 2016;7. 10.1128/mbio.02164-15. PMC4850275 27118586

[bib28] Fernandez De Landa G, Alberoni D, Baffoni L. et al. The gut microbiome of solitary bees is mainly affected by pathogen assemblage and partially by land use. Environ Microbiome. 2023;18:38. 10.1186/s40793-023-00494-w. PMC10131457 37098635

[bib29] Ferrari A, Tommasi N, Polidori C. Urbanisation reduced body size but potentially improved flight performance in bees and wasps. Basic Appl Ecol. 2024;74:57–65. 10.1016/j.baae.2023.11.010.

[bib30] Fitch G, Wilson CJ, Glaum p et al. Does urbanization favour exotic bee species? Implications for the conservation of native bees in cities. Biol Lett. 2019;15:20190274. 10.1098/rsbl.2019.0574. PMC6936029 31822245

[bib31] Gerth M, Saeed A, White JA. et al. Extensive screen for bacterial endosymbionts reveals taxon-specific distribution patterns among bees (Hymenoptera, Anthophila). FEMS Microbiol Ecol. 2015;91:fiv047. 10.1093/femsec/fiv047. 25914139

[bib32] Gholamy A, Kreinovich V, Kosheleva O. Why 70/30 or 80/20 relation between training and testing sets: a pedagogical explanation. Depart Tech Rep (CS). 2018:1209. https://scholarworks.utep.edu/cs_techrep/1209 (1 june 2025, date last accessed).

[bib33] Gibbs J, Hanuschuk E, Miller R. et al. A checklist of the bees (Hymenoptera: apoidea) of Manitoba, Canada. Can Entomol. 2023;155:1–38. 10.4039/tce.2022.45.

[bib34] Glöckner FO, Yilmaz P, Quast C. et al. 25 years of serving the community with ribosomal RNA gene reference databases and tools. J Biotechnol. 2017;261:169–76. 10.1016/j.jbiotec.2017.06.1198. 28648396

[bib35] Graystock P, Rehan SM, McFrederick QS. Hunting for healthy microbiomes: determining the core microbiomes of *Ceratina, Megalopta*, and *Apis* bees and how they associate with microbes in bee collected pollen. Conserv Genet. 2017;18:701. 10.1007/s10592-017-0937-7.

[bib36] Greenleaf SS, Williams NM, Winfree R. et al. Bee foraging ranges and their relationship to body size. Oecologia. 2007;153:589–96. 10.1007/s00442-007-0752-9. 17483965

[bib37] Grüter C, Segers FHID, Hayes L. Extensive loss of forage diversity in social bees owing to flower constancy in simulated environments. Proc R Soc B. 2024;291:20241036. 10.1098/rspb.2024.1036. PMC11289734 39082242

[bib38] Hammer Ø, Harper DAT, Ryan PD. PAST: paleontological statistics software package for education and data analysis. Palaeontol Electron. 2001;4:1–9.

[bib39] Hammer TJ, Le E, Moran NA. Thermal niches of specialized gut symbionts: the case of social bees. Proc R Soc B. 2021;288:20201480. 10.1098/rspb.2020.1480. PMC7893241 33563119

[bib40] Hettiarachchi A, Cnockaert M, Joossens M. et al. The wild solitary bees *Andrena vaga, Anthophora plumipes, Colletes cunicularius*, and *Osmia cornuta* microbiota are host specific and dominated by endosymbionts and environmental microorganisms. Microb Ecol. 2023;86:3013–26. 10.1007/s00248-023-02304-9. 37794084

[bib41] Hirose S, Tanda S, Kiss L. et al. Molecular phylogeny and evolution of the maple powdery mildew (*Sawadaea*, Erysiphaceae) inferred from nuclear rDNA sequences. Mycol Res. 2005;109:912–22. 10.1017/S0953756205003527. 16175793

[bib42] Hotchkiss MZ, Poulain AJ, Forrest JRK. Bumble bee gut microbial community structure differs between species and commercial suppliers, but metabolic potential remains largely consistent. Appl Environ Microbiol. 2025;91. 10.1128/aem.02036-24. PMC11921327 39912643

[bib43] Hubert J, Bicianova M, Ledvinka O. et al. Changes in the bacteriome of honey bees associated with the parasite *Varroa destructor*, and pathogens *Nosema* and *Lotmaria passim*. Microb Ecol. 2017;73:685–98. 10.1007/s00248-016-0869-7. 27730366

[bib44] Hui N, Liu X, Kotze DJ. et al. Ectomycorrhizal fungal communities in urban parks are similar to those in natural forests but shaped by vegetation and park age. Appl Environ Microbiol. 2017;83:e01797–17. 10.1128/AEM.01797-17. PMC5691411 28970220

[bib45] Kelly TT, Elle E. Investigating bee dietary preferences along a gradient of floral resources: how does resource use align with resource availability?. Insect Sci. 2021;28:555–65. 10.1111/1744-7917.12785. 32240575

[bib46] Khalifa SAM, Elshafiey EH, Shetaia AA. et al. Overview of bee pollination and its economic value for crop production. Insects. 2021;12:688. 10.3390/insects12080688. PMC8396518 34442255

[bib47] Kwong WK, Medina LA, Koch H. et al. Dynamic microbiome evolution in social bees. Sci Adv. 2017;3:e1600513. 10.1126/sciadv.1600513. PMC5371421 28435856

[bib48] Kwong WK, Moran NA. Gut microbial communities of social bees. Nat Rev Microbiol. 2016;14:374–84. 10.1038/nrmicro.2016.43. PMC5648345 27140688

[bib49] Kuhn M Building predictive models in R using the caret package. Journal of Statistical Software. 2008;28:1–26. 10.18637/jss.v028.i05

[bib50] Liaw A, Wiener M. Classification and regression by randomForest. R News. 2002;2:18–22.

[bib51] MacIvor JS. Building height matters: nesting activity of bees and wasps on vegetated roofs. Israel J Ecol Evol. 2015;62:88–96. 10.1080/15659801.2015.1052635.

[bib52] Martin M. CUTADAPT removes adapter sequences from high-throughput sequencing reads. EMBnet j. 2011;17:10. 10.14806/ej.17.1.200.

[bib53] Matteson KC, Grace JB, Minor ES. Direct and indirect effects of land use on floral resources and flower-visiting insects across an urban landscape. Oikos. 2012;122:682–94. 10.1111/j.1600-0706.2012.20229.x.

[bib54] McFrederick QS, Rehan SM. Characterization of pollen and bacterial community composition in brood provisions of a small carpenter bee. Mol Ecol. 2016;25:2302–11. 10.1111/mec.13608. 26945527

[bib55] McMurdie PJ, Holmes S. phyloseq: an R package for reproducible interactive analysis and graphics of microbiome census data. PLoS One. 2013;8:e61217. 10.1371/journal.pone.0061217. PMC3632530 23630581

[bib56] Melanson JB, Kelly TT, Waters S. et al. Agricultural intensification shapes community composition and parasite prevalence of native but not introduced bumble bees. Basic Appl Ecol. 2026;96:151836, 10.1016/j.baae.2026.06.008.

[bib57] Mesquita-Neto JN, Blüthgen N, Schlindwein C. Flowers with poricidal anthers and their complex interaction networks—disentangling legitimate pollinators and illegitimate visitors. Funct Ecol. 2018;32:2321–32. 10.1111/1365-2435.13204.

[bib58] Mikát M, Černá K, Straka J. Major benefits of guarding behavior in subsocial bees: implications for social evolution. Ecol Evol. 2016;6:6784–97. 10.1002/ece3.2387. PMC5513229 28725359

[bib59] Mitchell TB. Bees of the eastern United States V1. North Carolina Agricultural Experiment Station Technical. Bull Am Assoc Hist Nurs. 1960;141:1–538.

[bib60] Mitchell TB. Bees of the eastern United States V2. North Carolina Agricultural Experiment Station Technical. Bull Am Assoc Hist Nurs. 1962;152: 1–557.

[bib61] Motta EVS, Moran NA. The honeybee microbiota and its impact on health and disease. Nat Rev Microbiol. 2024;22:122–37. 10.1038/s41579-023-00990-3. PMC10998682 38049554

[bib62] Motta EVS, Seong J, Dickey M. et al. Divergent microbiome signatures between managed and wild honey bee (*Apis mellifera*) populations in South Texas. Microbiol Spectr. 2026;14:e03394–25. 10.1128/spectrum.03394-25. PMC12955401 41609372

[bib63] NatureServe . NatureServe network biodiversity location data accessed through NatureServe Explorer [web application]. Arlington: NatureServe, 2025. https://explorer.natureserve.org/. (10 July 2025, date last accessed).

[bib64] Nguyen PN, Rehan SM. The effects of urban land use gradients on wild bee microbiomes. Front Microbiol. 2022;13. 10.3389/fmicb.2022.992660. PMC9714450 36466654

[bib65] Nguyen PN, Samad-zada F, Chau KD. et al. Microbiome and floral associations of a wild bee using biodiversity survey collections. Environ Microbiol. 2024;26:e16657. 10.1111/1462-2920.16657. 38817079

[bib66] Ogle DH, Doll JC, Wheeler AP. et al. FSA: simple fisheries stock assessment methods. R package version 0.9.5. CRAN, 2023.

[bib67] Oksanen J, Blanchet FG, Friendly M. et al. Package ‘vegan’. Community Ecology Package version 2.5-7. CRAN, 2020.

[bib68] Ostwald MM, Gonzalez VH, Chang C. et al. Toward a functional trait approach to bee ecology. Ecol Evol. 2024;14:e70465. 10.1002/ece3.70465. PMC11487340 39429800

[bib69] Paluszynska A, Biecek P, Jiang Y. randomForestExplainer: explaining and visualizing random forests in terms of variable importance. R package version 0.10.1. CRAN, 2020.

[bib70] Pei CK, Hovick TJ, Limb RF. et al. Native and introduced pollinators vary in their seasonal floral resource visitation and selection between native and exotic plant species. J Appl Ecol. 2023;60:1424–34. 10.1111/1365-2664.14416.

[bib71] Peters B, Leonhardt SD, Schloter M. et al. Direct and indirect effects of land use on microbiomes of trap-nesting solitary bee larvae and nests. Front Microbiol. 2025;15. 10.3389/fmicb.2024.1513096. PMC11753253 39845038

[bib72] QGIS.org . QGIS Geographic Information System. Laax: QGIS Association, 2024. http://www.qgis.org.

[bib73] Qin W, Chen P, He R. Study on the dynamic distribution of spores of powdery mildew pathogen in wheat by rotor airflow of plant protection UAV. PLoS One. 2023;18:e0288248. 10.1371/journal.pone.0288248. PMC10629645 37934777

[bib74] Quast C, Pruesse E, Yilmaz P. et al. The SILVA ribosomal RNA gene database project: improved data processing and web-based tools. Nucleic Acids Res. 2013;41:D590–6. 10.1093/nar/gks1219. PMC3531112 23193283

[bib75] Raiol RL, Gastauer M, Campbell AJ. et al. Specialist bee species are larger and less phylogenetically distinct than generalists in tropical plant-bee interaction networks. Front Ecol Evol. 2021;9. 10.3389/fevo.2021.699649.

[bib76] R Core Team . R: a language and environment for statistical computing. Vienna: R Foundation for Statistical Computing, 2021. https://www.R-project.org./.

[bib77] Rutkowski D, Weston M, Vannette RL. Bees just wanna have fungi: a review of bee associations with nonpathogenic fungi. FEMS Microbiol Ecol. 2023;99:fiad077. 10.1093/femsec/fiad077. PMC10370288 37422442

[bib78] Sayers E. The E-utilities in-Depth: parameters, Syntax and More. In: Entrez Programming Utilities Help. Bethesda: National Center for Biotechnology Information (US), 2009. https://www.ncbi.nlm.nih.gov/books/NBK25499./ (30 November 2022, date last accessed).

[bib79] Sless TL, Nguyen PN, Chau KD. et al. Microbial communities of wild bees and comparative phylogenetics of key bacterial taxa across the bee tree of life. Proc R Soc B. 2025;293:20251823. 10.1098/rspb.2025.1823 41537975

[bib80] Stemkovski M, Pearse WD, Griffin SR et al. Bee phenology is predicted by climatic variation and functional traits. Ecol Lett. 2020;23:1589–98. 10.1111/ele.13583. 32812695

[bib81] Thamm M, Reiß F, Sohl L. et al. Solitary bees host more bacteria and fungi on their cuticle than social bees. Microorganisms. 2023;11:2780. 10.3390/microorganisms11112780. PMC10673014 38004791

[bib82] Todorov SD, Alves MV, Bueno GCA. et al. Bee-associated beneficial microbes—importance for bees and for humans. Insects. 2024;15:430. 10.3390/insects15060430. PMC11204305 38921144

[bib83] Turner TR, James EK, Poole PS. The plant microbiome. Genome Biol. 2013;14:209. 10.1186/gb-2013-14-6-209. PMC3706808 23805896

[bib84] Wilson CJ, Jamieson MA. The effects of urbanization on bee communities depends on floral resource availability and bee functional traits. PLoS One. 2019;14:e0225852. 10.1371/journal.pone.0225852. PMC6886752 31790482

[bib85] Ye M-H, Fan S-H, Li X-Y. et al. Microbiota dysbiosis in honeybee (*Apis mellifera* L.) larvae infected with brood diseases and foraging bees exposed to agrochemicals. R Soc Open Sci. 2021;8:201805. 10.1098/rsos.201805. PMC7890499 33614099

[bib86] Yilmaz P, Parfrey LW, Yarza P. et al. The SILVA and “All-species Living Tree Project (LTP)” taxonomic frameworks. Nucl Acids Res. 2014;42:D643–8. 10.1093/nar/gkt1209. PMC3965112 24293649

[bib87] Zhang ZJ, Huang MF, Qiu LF. et al. Diversity and functional analysis of Chinese bumblebee gut microbiota reveal the metabolic niche and antibiotic resistance variation of *Gilliamella*. Insect Sci. 2021;28:302–14. 10.1111/1744-7917.12770. 32101381

