# Supplementary material for: Variation in microbial and plant associations across wild bee families, functional groups, and urban environments
Source: FEMS Microbiol Ecol. 2026 Sep 30;102(10):fiag109. doi: 10.1093/femsec/fiag109 (PMC13643926; doi:10.1093/femsec/fiag109)
Supplement: Supplementary file 1 [file fiag109_supplemental_files.zip › Figure S1-Diversityplots_AlphaBeta_Genus.pdf]

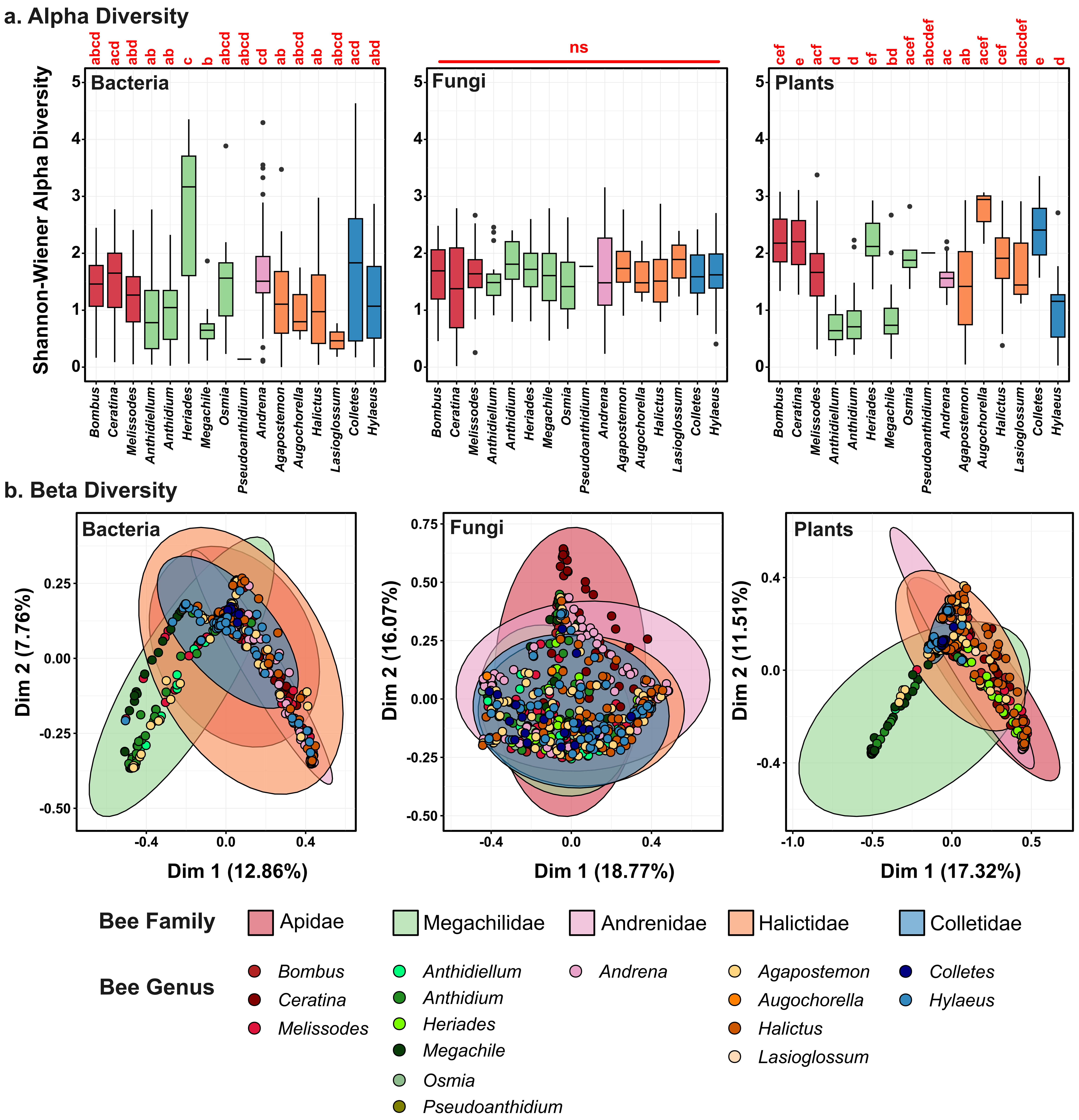

a. Alpha Diversity
Shannon-Wiener Alpha Diversity
Bacteria
Fungi
Plants
ns
abcd acd abd ab ab c b abcd abcd cd ab abcd ab abcd acd abd
cef e acf d d ef bd acef abcdef ac ab acef cef abcdef e d
Bombus
Ceratina
Melissodes
Anthidiellum
Anthidium
Heriades
Megachile
Osmia
Pseudoanthidium
Andrena
Agapostemon
Augochorella
Halictus
Lasioglossum
Colletes
Hylaeus
b. Beta Diversity
Bacteria
Dim 1 (12.86%)
Dim 2 (7.76%)
Fungi
Dim 1 (18.77%)
Dim 2 (16.07%)
Plants
Dim 1 (17.32%)
Dim 2 (11.51%)
Bee Family
Apidae
Megachilidae
Andrenidae
Halictidae
Colletidae
Bee Genus
Bombus
Ceratina
Melissodes
Anthidiellum
Anthidium
Heriades
Megachile
Osmia
Pseudoanthidium
Andrena
Agapostemon
Augochorella
Halictus
Lasioglossum
Colletes
Hylaeus
