# Supplementary material for: Variation in microbial and plant associations across wild bee families, functional groups, and urban environments
Source: FEMS Microbiol Ecol. 2026 Sep 30;102(10):fiag109. doi: 10.1093/femsec/fiag109 (PMC13643926; doi:10.1093/femsec/fiag109)
Supplement: Supplementary file 1 [file fiag109_supplemental_files.zip › Figure S2-Diversityplots_AlphaBeta_Species.pdf]

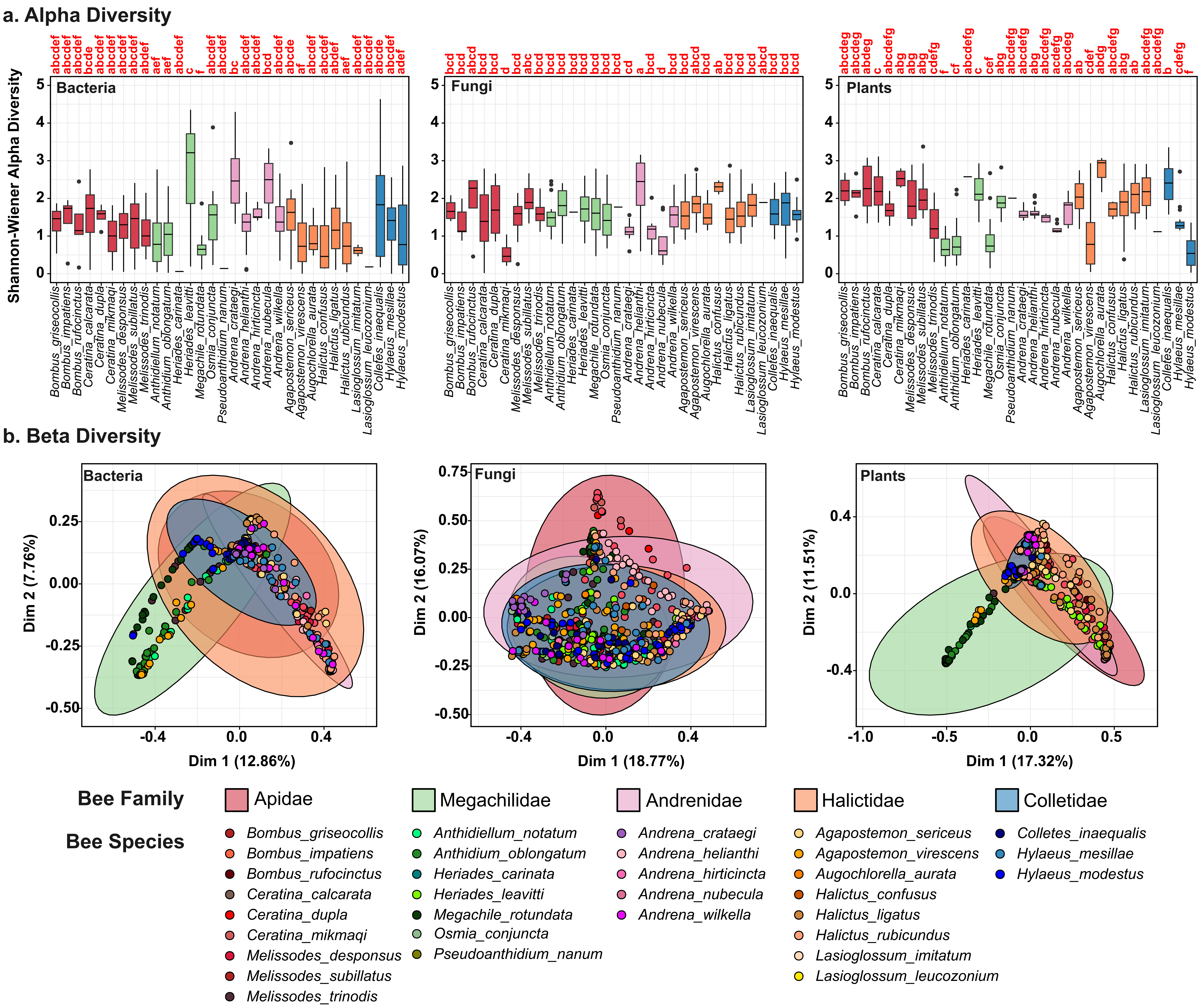

a. Alpha Diversity
Shannon-Wiener Alpha Diversity
Bacteria
Fungi
Plants
b. Beta Diversity
Bacteria
Fungi
Plants
Dim 1 (12.86%)
Dim 2 (7.76%)
Dim 1 (18.77%)
Dim 2 (16.07%)
Dim 1 (17.32%)
Dim 2 (11.51%)
Bee Family
Apidae
Megachilidae
Andrenidae
Halictidae
Colletidae
Bee Species
Bombus_griseocollis
Bombus_impatiens
Bombus_rufocinctus
Ceratina_calcarata
Ceratina_dupla
Ceratina_mikmaqi
Melissodes_desponsus
Melissodes_subillatus
Melissodes_trinodis
Anthidiellum_notatum
Anthidium_oblongatum
Heriades_carinata
Heriades_leavitti
Megachile_rotundata
Osmia_conjuncta
Pseudoanthidium_nanum
Andrena_crataegi
Andrena_helianthi
Andrena_hirticincta
Andrena_nubecula
Andrena_wilkella
Agapostemon_sericeus
Agapostemon_virescens
Augochlorella_aurata
Halictus_confusus
Halictus_ligatus
Halictus_rubicundus
Lasioglossum_imitatum
Lasioglossum_leucozonium
Colletes_inaequalis
Hylaeus_mesillae
Hylaeus_modestus
