# Supplementary material for: Variation in microbial and plant associations across wild bee families, functional groups, and urban environments
Source: FEMS Microbiol Ecol. 2026 Sep 30;102(10):fiag109. doi: 10.1093/femsec/fiag109 (PMC13643926; doi:10.1093/femsec/fiag109)
Supplement: Supplementary file 1 [file fiag109_supplemental_files.zip › Figure S3-Diversityplots_AlphaBeta_Functional.pdf]

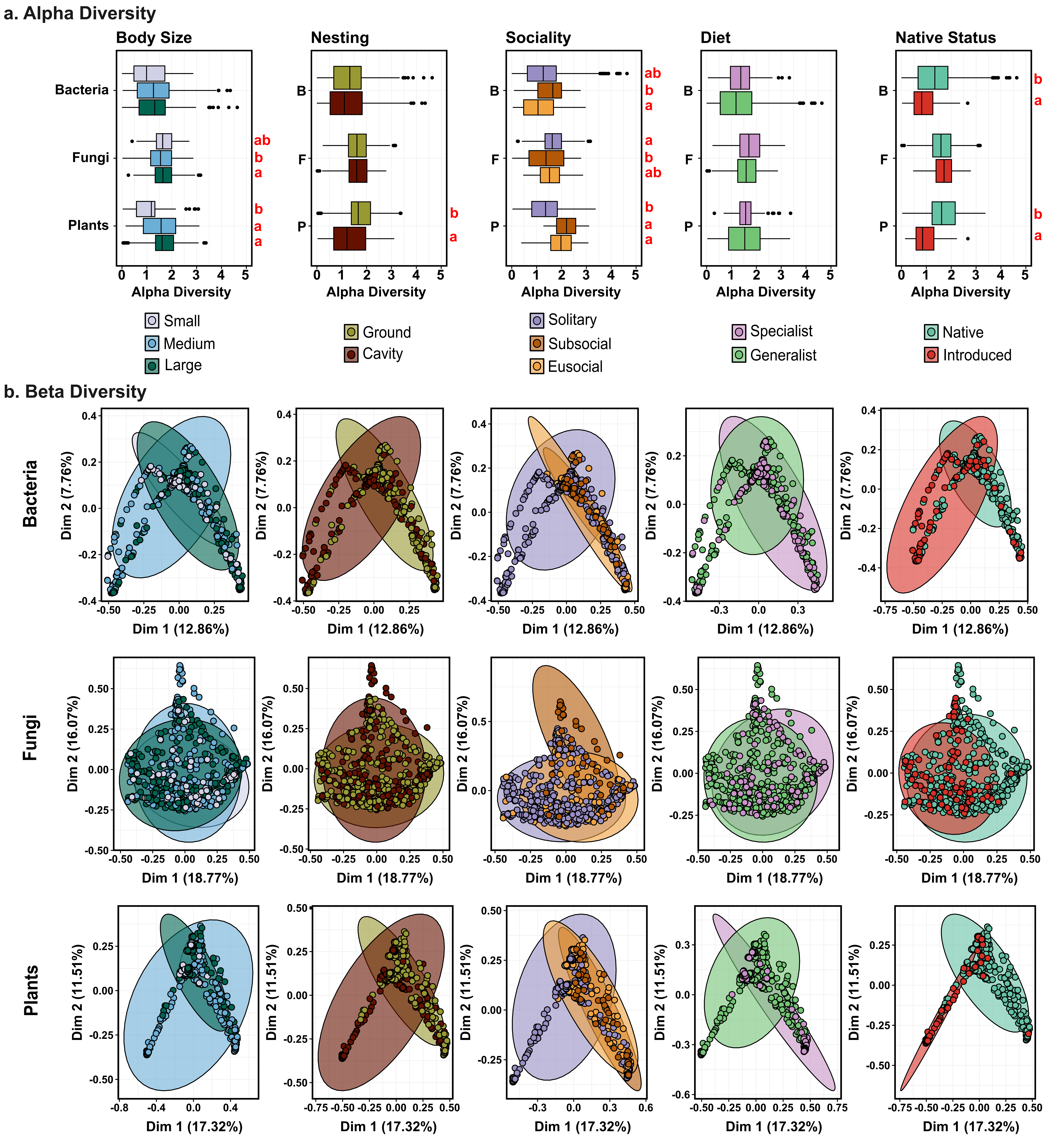
a. Alpha Diversity
Body Size
Nesting
Sociality
Diet
Native Status
Bacteria
Fungi
Plants
B
F
P
ab
b
a
Alpha Diversity
Small
Medium
Large
Ground
Cavity
Solitary
Subsocial
Eusocial
Specialist
Generalist
Native
Introduced
b. Beta Diversity
Bacteria
Fungi
Plants
Dim 1 (12.86%)
Dim 2 (7.76%)
Dim 1 (18.77%)
Dim 2 (16.07%)
Dim 1 (17.32%)
Dim 2 (11.51%)
