# Supplementary material for: Variation in microbial and plant associations across wild bee families, functional groups, and urban environments
Source: FEMS Microbiol Ecol. 2026 Sep 30;102(10):fiag109. doi: 10.1093/femsec/fiag109 (PMC13643926; doi:10.1093/femsec/fiag109)
Supplement: Supplementary file 1 [file fiag109_supplemental_files.zip › Figure S4 - RelAbunTopTaxa - UPDATED.pdf]

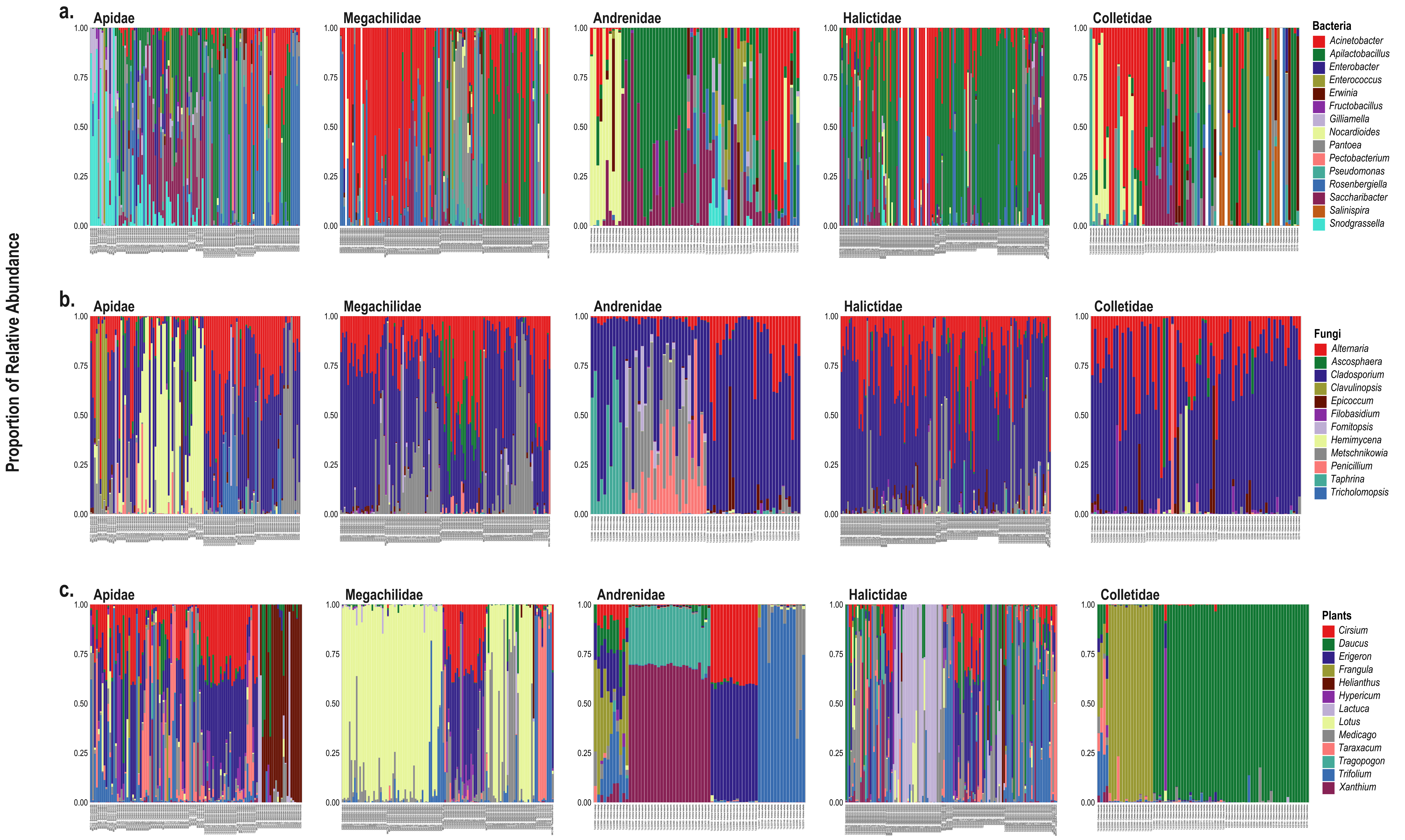

Proportion of Relative Abundance
a.
b.
c.
Apidae
Megachilidae
Andrenidae
Halictidae
Colletidae
1.00
0.75
0.50
0.25
0.00
Bacteria
Acinetobacter
Apilactobacillus
Enterobacter
Enterococcus
Erwinia
Fructobacillus
Gilliamella
Nocardioides
Pantoea
Pectobacterium
Pseudomonas
Rosenbergiella
Saccharibacter
Salinispira
Snodgrassella
Fungi
Alternaria
Ascosphaera
Cladosporium
Clavulinopsis
Epicoccum
Filobasidium
Fomitopsis
Hemimycena
Metschnikowia
Penicillium
Taphrina
Tricholomopsis
Plants
Cirsium
Daucus
Erigeron
Frangula
Helianthus
Hypericum
Lactuca
Lotus
Medicago
Taraxacum
Tragopogon
Trifolium
Xanthium
