# Supplementary material for: Variation in microbial and plant associations across wild bee families, functional groups, and urban environments
Source: FEMS Microbiol Ecol. 2026 Sep 30;102(10):fiag109. doi: 10.1093/femsec/fiag109 (PMC13643926; doi:10.1093/femsec/fiag109)
Supplement: Supplementary file 1 [file fiag109_supplemental_files.zip › Figure S5-CommonTaxa.pdf]

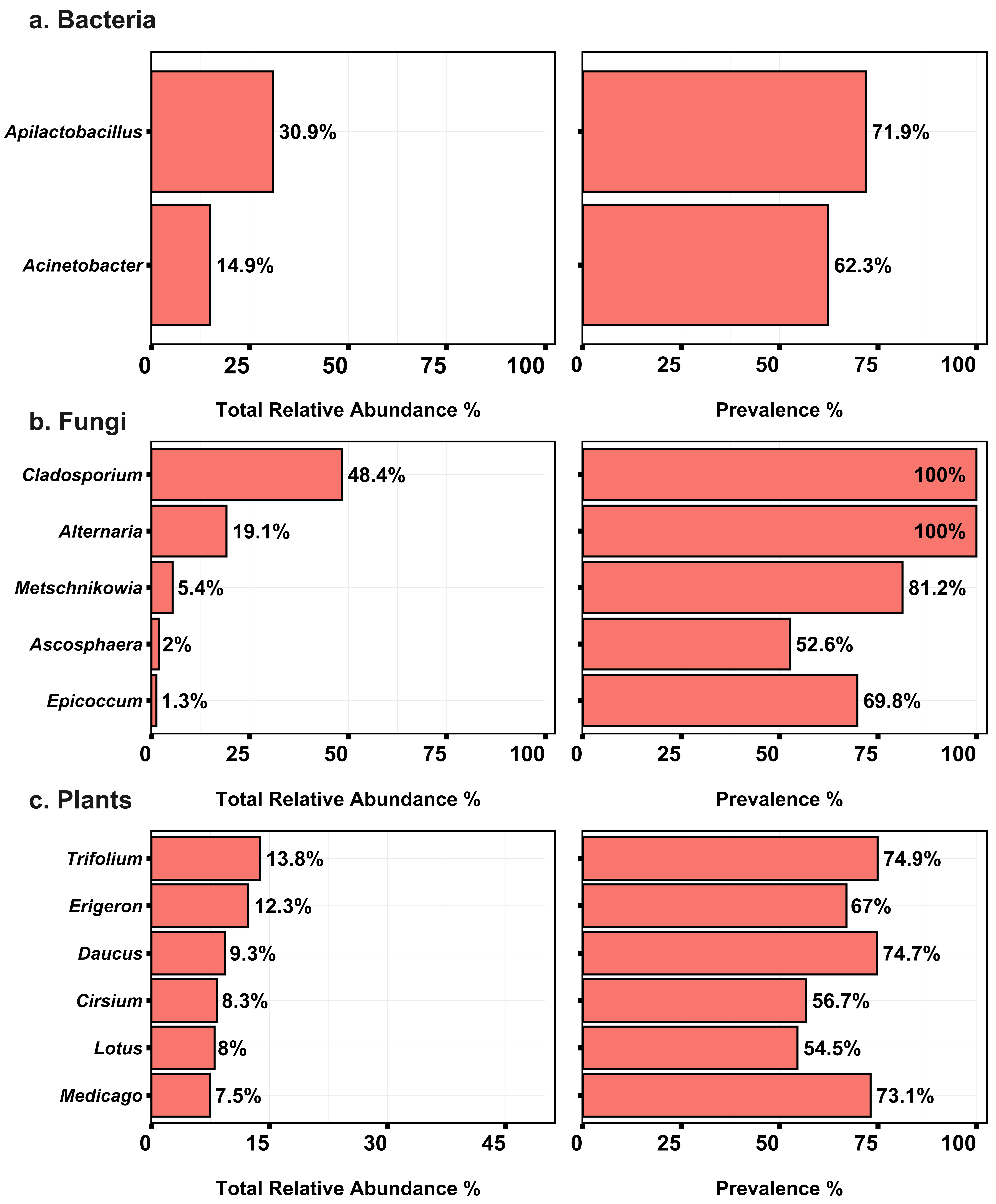
a. Bacteria
Apilactobacillus
Acinetobacter
30.9%
14.9%
71.9%
62.3%
0
25
50
75
100
Total Relative Abundance %
Prevalence %
b. Fungi
Cladosporium
Alternaria
Metschnikowia
Ascosphaera
Epicoccum
48.4%
19.1%
5.4%
2%
1.3%
100%
100%
81.2%
52.6%
69.8%
Total Relative Abundance %
Prevalence %
c. Plants
Trifolium
Erigeron
Daucus
Cirsium
Lotus
Medicago
13.8%
12.3%
9.3%
8.3%
8%
7.5%
74.9%
67%
74.7%
56.7%
54.5%
73.1%
0
15
30
45
Total Relative Abundance %
Prevalence %
